# Performance and Characteristics of Wearable Sensor Systems Discriminating and Classifying Older Adults According to Fall Risk: A Systematic Review

**DOI:** 10.3390/s21175863

**Published:** 2021-08-31

**Authors:** Annica Kristoffersson, Jiaying Du, Maria Ehn

**Affiliations:** 1 School of Innovation, Design and Engineering, Mälardalen University, 722 20 Västerås, Sweden; annica.kristoffersson@mdh.se; 2 Motion Control i Västerås AB, 721 30 Västerås, Sweden; jiaying.du@motioncontrol.se

**Keywords:** fall risk, classification, assessment, older adults, inertial sensors, wearable sensors

## Abstract

Sensor-based fall risk assessment (SFRA) utilizes wearable sensors for monitoring individuals’ motions in fall risk assessment tasks. Previous SFRA reviews recommend methodological improvements to better support the use of SFRA in clinical practice. This systematic review aimed to investigate the existing evidence of SFRA (discriminative capability, classification performance) and methodological factors (study design, samples, sensor features, and model validation) contributing to the risk of bias. The review was conducted according to recommended guidelines and 33 of 389 screened records were eligible for inclusion. Evidence of SFRA was identified: several sensor features and three classification models differed significantly between groups with different fall risk (mostly fallers/non-fallers). Moreover, classification performance corresponding the AUCs of at least 0.74 and/or accuracies of at least 84% were obtained from sensor features in six studies and from classification models in seven studies. Specificity was at least as high as sensitivity among studies reporting both values. Insufficient use of prospective design, small sample size, low in-sample inclusion of participants with elevated fall risk, high amounts and low degree of consensus in used features, and limited use of recommended model validation methods were identified in the included studies. Hence, future SFRA research should further reduce risk of bias by continuously improving methodology.

## 1. Introduction

Falls are the second leading cause of accidental or unintentional injury resulting in death worldwide [1]. Approximately 35% of all people aged 65 years or older fall every year [2] and the incidence of falls increases with age [3]. Important risk factors include impaired balance and gait performance, polypharmacy, and a history of previous falls [4]. Interventions combining fall preventive physical activities with strategies to increase safety in home environments have proven to be the most effective in reducing the incidence and risk of falls [5]. Technologies can improve fall prevention interventions’ efficiency and effectiveness. Hence, fall prevention technologies are mainly used to assess and decrease fall risk, to increase adherence to fall prevention training interventions or to detect occurring falls and alarms in case of an accident [6].

Sensor-based fall risk assessment (SFRA) utilizes wearable sensors for monitoring individuals’ motions during assessment tasks. The sensor signals are processed, and specific features are extracted and incorporated into algorithms which aim at predicting fall occurrence or classifying individuals into risk categories [7]. Several reviews of state-of-the-art of SFRA research were published during 2012–2019. 

In 2012, Shany et al. discussed the practicalities and challenges associated with the use of wearable sensors for the quantification of older people’s fall risk [7]. They identified several study design elements that need to be fulfilled in order to support future real-life use of SFRA. These include: (1) prospective design, (2) larger validations of higher quality enabling meta-analyses, (3) rigorous testing including reliability of test-retest and rater-effects, (4) validation of SFRA-tools on different samples and by research groups other than those suggesting/developing the tool, and (5) an increased focus on SFRA supporting clinical staff in supervised assessments [7].

The following year, Howcroft et al. (2013) made a systematic review of SFRA in geriatric populations using inertial sensors. The review was based on 40 articles published 2003–March 2013 and confirmed the need of prospective design in SFRA research [8]. Moreover, Howcroft et al. emphasized the need to use separate datasets in training and validation of classification models, and more appropriate intelligent computing methods, such as neural networks and Bayesian classifiers, instead of regression [8]. The use of separate datasets had been neglected in 50% of the studies involving classification models included in the review [8]. Howcroft et al. also identified a need for: (1) systematically assessing which combinations of body locations of sensors and sensor-based variables result in high reliability, (2) investigating long-term user compliance to SFRA methods, (3) using SFRA in specialized populations, systematic matching of predictive variables and specific fall risk factors, and (4) comparing accuracies of SFRA methods with accuracies of clinical assessments, both obtained by prospective studies [8].

In 2015, Shany et al. published a review of articles including features extracted from sensor signals in statistical models intended to estimate fall risk or predict falls in older people [9]. This review, which was based on 31 articles published 1997–2015, identified problems with publication bias, inadequate sample sizes, inadequate number of fall events in samples, misuse and lack of model validation, deficiencies in model selection and feature extraction procedures, and insufficient use of prospective fall occurrence as serious issues [9]. Shany et al. (2015) pointed out that some of the included studies reported classification accuracies exceeding the estimated theoretical maximal accuracy (0.81) in predicting the occurrence of a fall during a one-year period [10]. They concluded that the prediction performance was overestimated in the literature, mainly due to small samples, large feature pools, model overfitting, lack of validation, and misuse of modelling techniques [9]. Therefore, Shany et al. suggested that sample bias should be prevented by recruiting cohorts ensuring that an adequate number of falls occur and by considering the recommendations of 1:10 features/event [11] during feature selection [9]. They also suggested improvements in feature selection by tightening the significance thresholds, removing redundant features, and selecting the correct statistical methods [9]. Finally, the need for appropriate model validation methods, preferably by external validation of the final model, was stressed [9].

Roeing et al. [12] conducted a review on the use of mobile health applications for assessment of balance, i.e., one of the fall risk factors. The article included 13 articles published 2011–2016. Several of the articles included young samples, while others lacked information on the studied group of participants. Five articles assessed the validity of mobile health applications by comparing the data collected with data collected using 3D motion capture measurement, an accelerometer or a force platform.

Three systematic reviews were published in 2018 [13,14,15]. Sun and Sosnoff [13] reviewed the use of novel sensing technology in fall risk assessment in 22 articles published 2011–May 2017. Their recommendations for future research included the use of: (1) prospective fall occurrence of at least 6 months to label subjects, (2) a reduced number of variables, selection of variables based on previous research, and (3) appropriate model validation [13].

Montesinos et al. [14] presented a systematic review and meta-analysis of the use of wearable inertial sensors for fall risk assessment and prediction in older adults. The review included 13 articles published up until 2016. Montesinos et al. [14] identified strong/very strong associations between fall risk assessment outcomes and nine triads (combinations of a sensor feature category, a task, and a sensor placement). The recommended and not-recommended triads were found to be task-dependent when analyzing the tasks quiet standing, sit-to-stand/stand-to-sit, Timed Up and Go (TUG) test and walking. For both quiet standing and sit-to-stand/stand-to-sit, the recommended feature category and sensor location were linear acceleration and lower back. For TUG, the recommended sensor category and sensor location were temporal and shins. For walking, there existed both recommended and not-recommended triads. The recommended combinations of sensor feature category and sensor location for walking task were: (1) angular velocity-shins, (2) frequency-upper back, and (3) frequency-lower back. The not-recommended combinations of sensor category and sensor location during walking were: (1) angular velocity-lower back, (2) frequency-shins, and (3) linear acceleration-shins. Hence, the sensor location recommended by [14] varies depending on the feature category, particularly for walking.

Rucco et al. [15] reviewed the type and location of wearable sensors for monitoring falls during static and dynamic tasks in healthy elderly. The review was based on 42 articles published 2002–2017. Rucco et al. concluded that the majority of studies used a maximum of two sensors with accelerometers and gyroscopes being the most common, and that the majority of studies presented preliminary results [15]. The trunk was identified as the most studied body segment. The most frequently used tasks varied depending on whether the task was static or dynamic. For measuring static stability, a quiet standing test with eyes opened/closed was most common. For dynamic evaluations, the most common tasks were walking and stand-sit tests [15]. Finally, Rucco et al. [15] stated that information on performance, i.e., accuracy, sensitivity, and specificity, was too diverse and did not allow for evaluating the impact of different system characteristics. Therefore, they identified the need for golden standards in terms of sensors (types, position) and tasks.

In 2019, Bet et al. made a systematic review on fall detection and fall risk assessment in older persons using wearable sensors [16]. The review, which was based on 29 different articles published 2002–2019, presented performance metrics and reported on number of sensors, sensor types, sensor location and assessment tasks. It should be noted that 20 of the articles included only accelerometer features. The use of other sensors was sparse, one article used only gyroscope features, five used a combination of accelerometer and gyroscope features, two used a combination of accelerometer and barometer features, and one used a combination of accelerometer, gyroscope, and magnetometer features. Bet et al. also analyzed sensor locations and found that the most common location was the waist (8 articles), followed by the lumbar region (7), ankle (4), pelvis (4), and head (3) [16].

It is worth noticing here that different terminologies have potentially been used to denote the same sensor location in previous review articles. For example, Montesinos et al. [14], who identified recommended and not-recommended triads, used the notation shins in their triads, while Bet et al. [16] identified four articles with sensors located on the ankle. Further, the most frequently used locations in [16] were the waist and lower back (lumbar spine) whereas [14] stated that the most common placement was the lower back (approximately L3). Rucco et al. [15] used the notation trunk for sensors located at L3, L5, sternum, waist, pelvis, neck, and chest. Hence, comparing the results obtained in this review with results from the previous reviews is not straightforward.

The aim of this systematic review was to analyze the characteristics and performance of wearable sensor systems used to assess older people’s fall risk by classifying individuals according to fall risk or by discriminating between groups of older people with different fall risk. The following research questions were in focus:

RQ1 What is the evidence of SFRA in terms of (a) discriminative capability, and (b) classification performance?
RQ2 Which of the previously identified risk factors for study bias can be identified among the included studies? The risk factors analyzed included: (a) low use of prospective study design, (b) use of small study samples with low amounts of fall events, (c) low consensus in features used in SFRA models; and (d) misuse of model validation methods.

## 2. Materials and Methods

### 2.1. Literature Search

The systematic literature review was conducted according to the PRISMA guidelines [17]. The review elements (aim including PICO elements, eligibility criteria and outcomes) are defined in Table 1.

The systematic literature search was done in four databases: Web of Science Core Collection (i.e., SCI-EXPANDED, SSCI, A&HCI, CPCI-S, CPCI-SSH and ESCI), IEEE Xplore, Pubmed, and Medline. Search phrases and search dates for each database are presented in Table 2. Web of Science and IEEE Xplore were searched twice with modifications made in search phrases.

### 2.2. Study Selection

**Figure 1:**
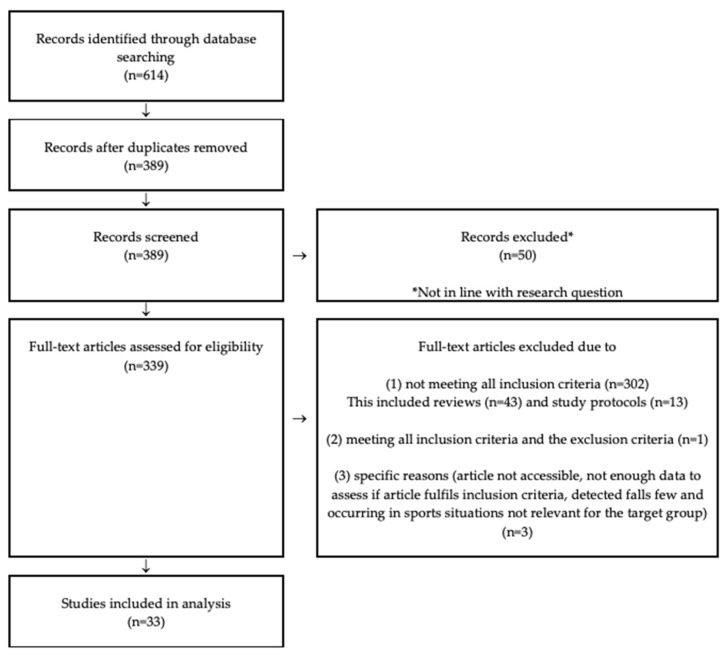
Flow diagram of articles identified and evaluated based on eligibility criteria.

The systematic literature search in the four databases identified 614 records. After removal of 225 duplicates, 389 publications were screened for eligibility according to the inclusion and exclusion criteria in Table 1. The titles and abstracts of potentially relevant articles were screened independently by two researchers (ME and AK). Eligibility assessments of full text records were performed independently by the same two researchers. In both steps, disagreement was resolved through discussions until consensus was reached. A total of 304 articles were excluded in the screening. Full text copies were downloaded for the remaining 33 articles included in this review (Figure 1).

### 2.3. Data Extraction

Data from the full text articles was extracted to a study specific template with defined variables (Table A1 in Appendix A). Data extraction was performed independently by three researchers (ME, AK, and JD). All reported data/results were discussed by at least two researchers until consensus was reached.

### 2.4. Research Questions and Data Analysis

The study’s two main research questions were:

RQ1 What is the evidence of SFRA in terms of (a) discriminative capacity, and (b) classification performance?
RQ2 Which of the previously identified risk factors for study bias can be identified among the included studies? The risk factors analyzed included: (a) low use of prospective study design, (b) use of small study samples with low amounts of fall events, (c) low consensus in features used in SFRA models, and (d) misuse of model validation methods.

In order to guide the analysis of the collected data, a larger number of more detailed research questions were formulated. These questions guided how the data, collected in the study specific data collection template (Appendix A), were summarized and presented in eight tables. Table 3 and Section 3.1 present data on study characteristics; Tables 4–6 and Section 3.2 and Section 3.3 present data on fall risk assessment system characteristics. Table 4 presents articles performing discrimination by feature selection. Tables 5 and 6 present articles performing classification methods/models with and without machine learning algorithms. Section 3.4 presents the results of an analysis on whether Montesinos et al.’s triads [14] can be identified in the included articles and whether the triad theory applies also on articles using classification models. Tables 7–10 and Section 3.5 present data on the evaluation methodology and fall risk discrimination/classification performance.

Qualitative data were analyzed according to content and quantitative data were analyzed using descriptive statistics if possible.

## 3. Results

The presented results include study characteristics (Section 3.1), wearable sensors used for fall risk assessment (Section 3.2), signal processing (Section 3.3), the identification of triads and assessment of their applicability on classification methods/models (Section 3.4), and statistical analysis on the sensor-based methods’ capabilities to assess fall risk (Section 3.5).

The studies were published between January 2010 and December 2019. The number of articles per year was highest in 2017 (*n* = 8) followed by 2011, 2016, 2018, and 2019 (*n* = 5), and 2014 (*n* = 3). Only one article from 2013 and 2015 respectively is included. None of the included articles were published in 2010, 2012, or 2020.

### 3.1. Study Characteristics

The characteristics of the 33 included studies are presented in Table 3.

#### 3.1.1. Authors Involved and Places Where the Research Was Conducted

The 33 included articles were authored by 145 authors affiliated in 16 countries on four continents (Asia, Europe, North America, and Oceania). Five authors were affiliated with organizations in two different countries. Most authors (116/145) authored one article. However, 21 authored two articles and eight authors were in the author list of at least four articles (number of articles in parenthesis): Brodie (4), Caulfield (4), Delbaere (4), Greene (6), Hausdorff (5), Lord (4), Redmond (4), and Weiss (4).

Most articles (25/33) were written by authors affiliated in the same country. The distribution per continent was as follows: Asia: Israel (*n* = 1), Japan (*n* = 2), South Korea (*n* = 1); Europe: Belgium (*n* = 1), Germany (*n* = 1), Ireland (*n* = 4), Italy (*n* = 1); North America: Canada (*n* = 2), United States (*n* = 6): and Oceania: Australia (*n* = 6). Eight articles had authors affiliated in different countries: Australia–Ireland (*n* = 1), Ireland–USA (*n* = 1), Israel–Taiwan-USA (*n* = 1), Israel–Norway (*n* = 1), Germany–Israel-Norway (*n* = 1), Czech Republic–France–Italy (*n* = 1), Belgium–Netherlands (*n* = 1), Belgium–Israel–Italy–Netherlands–UK–USA (*n* = 1). It is worth noting that the Australian articles were authored by two groups, one group authoring [45,48], and another group authoring [25,28,35,41]. Greene was on the author list of all articles from Ireland and on the author list of the Australia–Ireland and Ireland–USA articles. The USA articles were almost exclusively written by different research groups although two authors were on the author list for 2/6 articles from USA. The articles including authors from Israel were mostly authored in collaboration with authors from other countries.

#### 3.1.2. Study Populations

**Figure 2:**
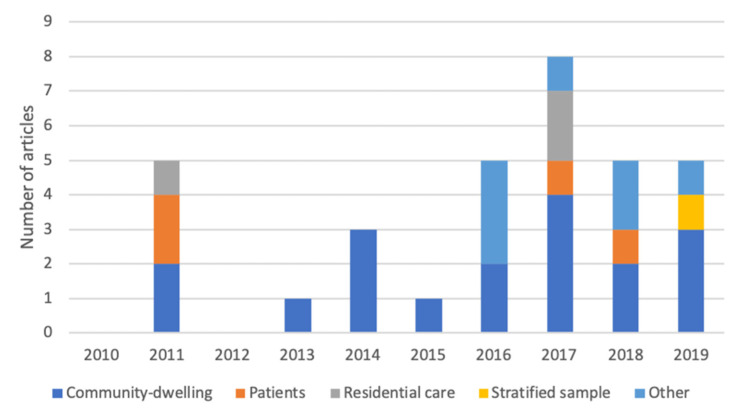
Number of studies per study population and publication year.

The study participants were classified as community-dwelling (18 articles), patients (four articles), residential care/continuing-care retirement community (two articles), and other (eight articles) if none of the aforementioned labels matched the reported population (e.g., “people from cohort,” or “convenience sample”). In addition, one study [49] had a large, stratified sample including subgroups of community-dwelling, residential care, and patients (neurological and rehabilitation). The populations of all studies per publication year are presented in Figure 2. Community-dwelling was the most studied population, and none of the other populations were studied in publications from 2013–2015. No other clear trends could be identified among the included studies in study population.

#### 3.1.3. Faller/Non-Faller (or Equivalent) Labelling Method

The studies generated outcomes to compare SFRAs by labelling the participants according to their fall risk. Although the most common outcome was non-faller/faller, other labels, namely frail/non-frail [37], low risk/at-risk [38,43,44] were also used. Moreover, [42,48,51] used three labels (non-faller/faller/multiple-faller).

**Figure 3:**
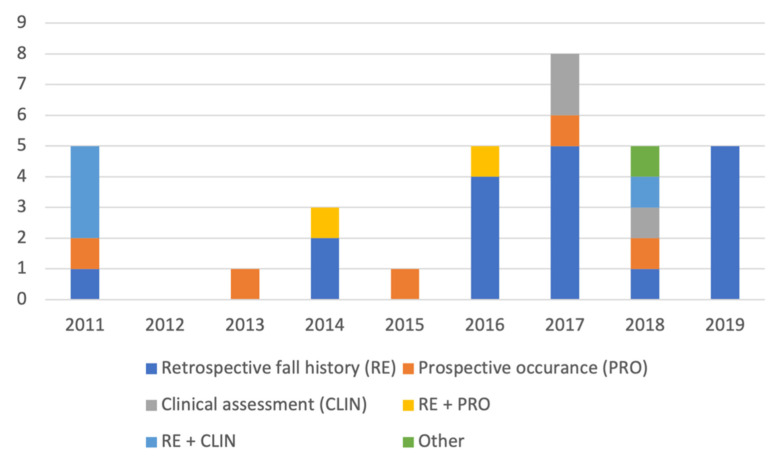
Number of studies per faller/non-faller (or equivalent) labelling method and publication year.

The most common method to label a participant as faller or a non-faller (or equivalent) was RE data alone (*n* = 18) or in combination with CLIN data (*n* = 4). Three of the included studies solely used CLIN data (formulas or functional tests) to label participants. However, two of these studies used clinical formulas which included RE data. Five studies used PRO data alone and two studies combined RE and PRO data (one of them compared performance of retrospective and prospective classification models [27]). Finally, one study [44] stated that clinical partners determined whether a participant was labelled as high fall risk or age matched low fall risk. This technique was categorized as “other” in the current review (Figure 3 and Table 4). Figure 3 presents the number of studies per publication year that applied the respective faller/non-faller (or equivalent) labelling method. As can be seen here, the use of PRO data (alone or in combination with RE data) had not increased during 2011–2019. In total, seven studies used PRO data, either alone or in combination with RE data, to label participants. PRO data was mainly followed up for 12 months (5/7 studies), although 6- and 24-months periods were also used. In total, 25 studies used RE data to label participants (either alone, in combination with PRO and CLIN data, or as part of CLIN data). RE data was mostly retrieved from the past 12 (16/25 studies), 60 (5/25 studies), 6 (2/25 studies), 3 (1/25 studies) or 18 months (1/25 studies). Moreover, one study did not specify the length of the period to collect RE data.

#### 3.1.4. Size and Proportion of Participants Labelled as Fallers of Study Samples

**Figure 4:**
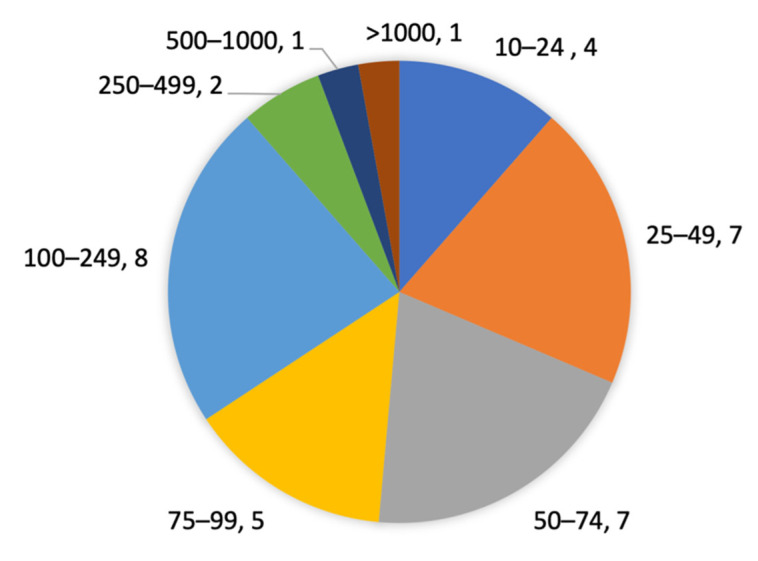
Distribution of study samples according to size.

The studies’ sample sizes ranged from 13 to 6295 participants (mean 289, median 73, standard deviation (SD) 1041). One study used three different datasets [27], which were counted as three separate samples in our analysis. One study published in 2019 [49] had an exceptionally large sample of 6295 participants. The studies were categorized into eight categories according to sample size. The distribution of studies for each categorized sample size is presented in Figure 4. Approximately one third (12/35) of the studies had a sample of at least 100 participants.

The proportion of participants labelled as having elevated fall risk (faller, frail or at risk) according to RE data (recorded during periods of 3–60 months) and/or PRO data (during periods of 6–24 months) and/or CLIN data ranged from 14% to 71% (mean 44%, median 46%, SD 14.7%), see Table 3. The threshold used to define a person with elevated fall risk (faller, frail or at risk) varied between the studies. For example, while most studies required at least one previous fall to label a participant as a faller in some studies, a few studies (pointed out in Table 3) required at least two falls. Moreover, most studies performed binary classification of participants (faller/non-faller) while a few studies classified participants into three groups (faller/once-faller/multiple faller). One of the study samples in [27] did not specify the percentage of fallers in sample, the sample was therefore omitted in the analysis. Moreover, one study used both RE and PRO data to label participants and obtained different proportion of fallers depending on method. Both values were included in the analysis.

#### 3.1.5. Sensor-Based Fall Risk Assessment Tasks and Degree of Supervision

Most of the studies (25/33) performed supervised SFRAs where assessment tasks tested walking (*n* = 9), sit-to-stand transitions in combination with walking (mostly in TUG) (*n* = 6), standing balance function (*n* = 4), sit-to-stand transitions (*n* = 2), turning balance (*n* = 1), choice stepping reaction time (*n* = 1), upper extremity function (*n* = 1), and TUG in combination with other clinical tests (*n* = 1). Two of the supervised tests were performed in a home setting. In two of the 33 studies, the SFRAs tasks (walking on flat surface and stairs and stair ascent) were performed in semi-supervised conditions at research facilities. Six of the 33 studies analyzed sensor data from unsupervised assessment tasks in a home environment, either ADL or free-living daily gait.

The number of different fall risk assessment tasks identified in this review was higher than the four tasks (quiet standing, sit-to-stand/stand-to-sit, TUG and walking) included in the triads identified in by [14].

Studies basing SFRA on classification methods/models with machine learning used fewer assessment than studies basing SFRA on feature selection and on classification models without machine learning. In addition, the use of unsupervised and semi-supervised assessments was higher among studies using classification methods/models with machine learning (50% supervised, 33% unsupervised and 17% semi-supervised) than among studies performing discrimination by feature selection (77% supervised, 18% unsupervised and 5% semi-supervised) and studies using classification models without machine learning (100% supervised).

### 3.2. Wearable Sensor Used for Fall Risk Assessment

This section provides an overview of trends in the number of wearable sensors and sensor types used, as well as the distribution of wearable sensors at different body locations. The identified differences between studies performing discrimination by feature selection and studies using classification methods/models with and without machine learning algorithms are presented.

#### 3.2.1. Number of Wearable Sensors

**Figure 5:**
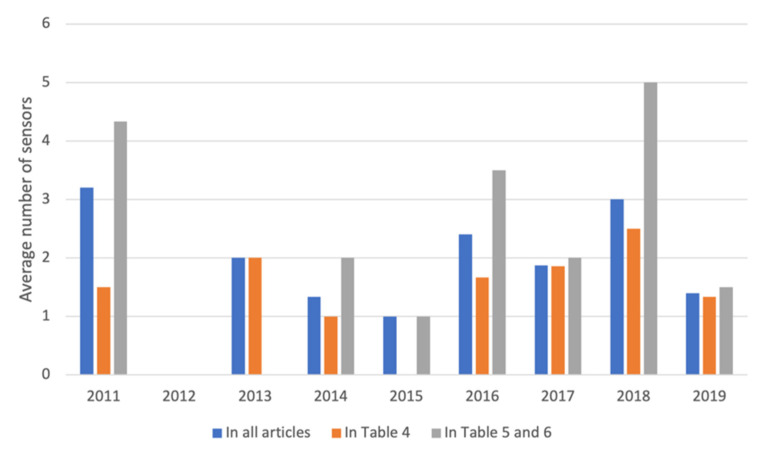
Average number of sensors per publication year for: (1) all articles, (2) the articles in Table 4, and (3) the articles in Table 5 and Table 6.

The average number of sensors per study varied between 1 and 5 among the articles. Most studies (26/33) used 1–2 sensors. As shown in Figure 5, the variation in average number of sensors used per article and year was higher for studies using classification methods/models (i.e., studies in Table 5 and Table 6 where the number varied between 1 and 10) than for studies not using classification methods/models (i.e., the studies in Table 4 where the number varied between 1 and 4). However, the difference in average number of sensors per publication year was not statistically significant between the two groups of studies.

#### 3.2.2. Sensor Types

This section provides information on different types of wearable sensors identified in the included articles, differences between article categories, and identified trends in sensor types.

The following sensor types were identified among the included studies (number of articles given in parenthesis): accelerometers (13), gyroscopes (5), a combination of accelerometers and gyroscopes (6), a combination of accelerometers, gyroscopes and magnetometers (3), a combination of accelerometers and barometer (2), a combination of accelerometers, gyroscopes, magnetometers and barometer (1), a combination of a 2D accelerometer and load cell (1), a combination of accelerometers and photoelectric heart rate (1), and a combination of accelerometers and pressure (1).

**Figure A1:**
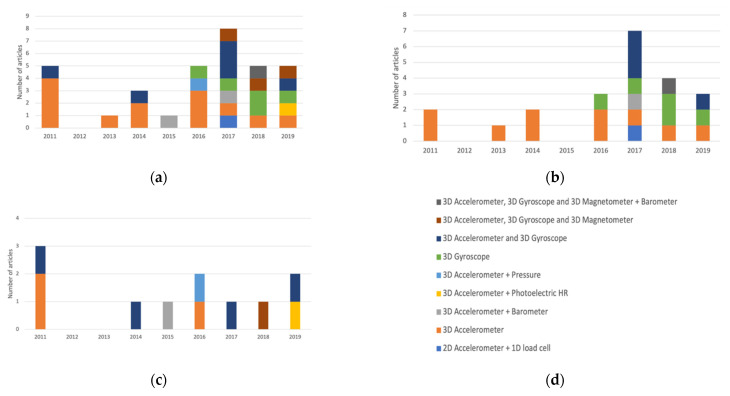
Information on number of articles using different types of sensors per publication year for: (**a**) all articles; (**b**) articles in Table 4; (**c**) articles in Table 5 and Table 6; and (**d**) the sensor types. HR = heart rate. Please note that HR is used for heart rate only in this figure.

During 2011–2019, the number of different sensor types used, i.e., their dimensionality, increased after 2016. As shown in Figure A1a (in Appendix B), all articles published during 2011–2015 used one or two different sensor types: accelerometers (7/10 articles), accelerometers and gyroscopes combined (2/10), as well as accelerometers and barometers (1/10) combined. Starting from 2016, the number of different sensor types has increased: articles published in 2016 used 3D accelerometers (3 articles), 3D gyroscope (1 article), as well as a combination of a 3D accelerometer and pressure sensor (1 article). During 2017–2019, the number of different sensor types continued to increase, and the dimensionality of the sensor systems increased to 9D (i.e., 3D accelerometers, 3D gyroscopes, 3D magnetometers) and even to 10D by adding barometer data as well.

The variation in number of different sensor types used was higher among the studies performing discrimination by feature selection (Table 4) than among studies using classification methods/models (Table 5 and Table 6). However, the difference was not statistically significant. As shown in Figure A1b (in Appendix B), 1–5 different sensor types were used per publication year among the articles performing discrimination by feature selection. Only 1–2 different sensor types were used per publication year among the studies using classification methods/models with or without machine learning (see Figure A1c in Appendix B). Moreover, the use of 3D accelerometers was higher among the studies performing discrimination by feature selection (see Table 4) than among the studies using classification methods/models (Table 5 and Table 6). None of the studies in Table 5 and Table 6 that were published during 2017–2019 used 3D accelerometers.

Among the identified studies performing discrimination by feature selection (Table 4), gyroscopes started to be used in 2016. In this period, gyroscopes were used to an equal extent as accelerometers. During 2018–2019, two studies using a combination of accelerometer, gyroscope, and magnetometer features were identified, one of them also combined with barometer features.

#### 3.2.3. Distribution of Wearable Sensors at Different Body Locations

This section provides information on how wearable sensors were distributed between body locations, whether there were differences between article categories, as well as trends in distribution.

**Figure A2:**
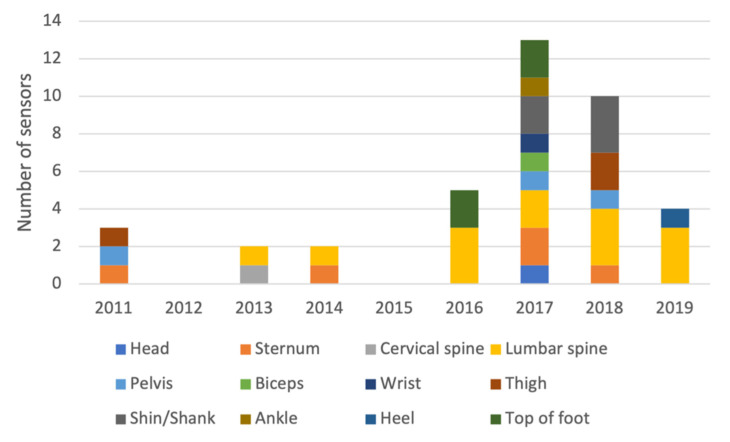
Number of sensors per body location for articles in Table 4, per publication year.

Starting with the studies performing discrimination by feature selection (i.e., the articles in Table 4), Figure A2 in Appendix B shows that a total of three sensors were used in the two articles from 2011. Two sensors were located on the upper body (pelvis and sternum) and one on the lower body (thigh). In 2013–2014, three articles used a total of four sensors, all located on the upper body (two on the lumbar spine, one on the cervical spine and one on sternum). In 2016, all five articles included sensors located on the upper body (lumbar spine) and one of them also included sensors located on the top of the feet. In 2017, seven articles used a total of nine different sensor body locations. Four of them had sensors located only on the upper body (pelvis, sternum, biceps, wrist, head). Two had sensors located both on the upper and lower body (in [39] at the sternum, lumbar spine, and on the feet, while in [41] on the lumbar spine and one of the ankles) and one [40] positioned the sensors on the shanks/shins. In 2018, all four articles used a sensor located on the upper body (three on the lumbar spine and one on the sternum and pelvis). In addition, two of them used sensors located on at least one shin/shank, and one of them used sensors located on the thighs. All three articles published in 2019 also used sensors located on the upper body (lumbar spine). In addition, one of them [50] used a sensor located on one of the heels.

**Figure 6:**
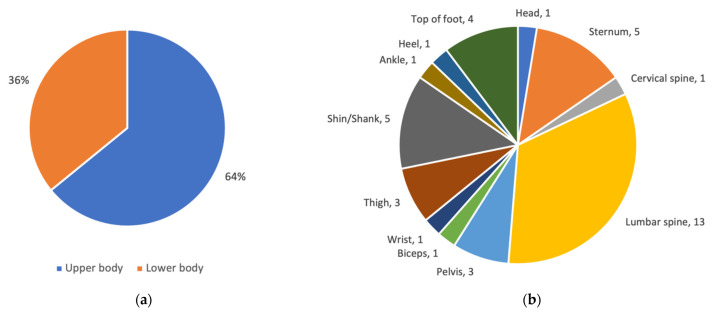
Information on sensor locations for articles in Table 4, i.e., the articles performing discrimination by feature selection: (**a**) Distribution of sensors located at the upper or lower body; (**b**) Number of sensors per body location.

To summarize, 64% of the wearable sensors used in the studies performing discrimination by feature selection were located on the upper body (Figure 6a). Figure 6b shows that the most common body location on the upper body was the lumbar spine (13 sensors). Other upper body locations used more than once include sternum (5), and pelvis (3). Lower body locations used more than once include shin/shank (5), top of foot (4), and thigh (3).

**Figure A3:**
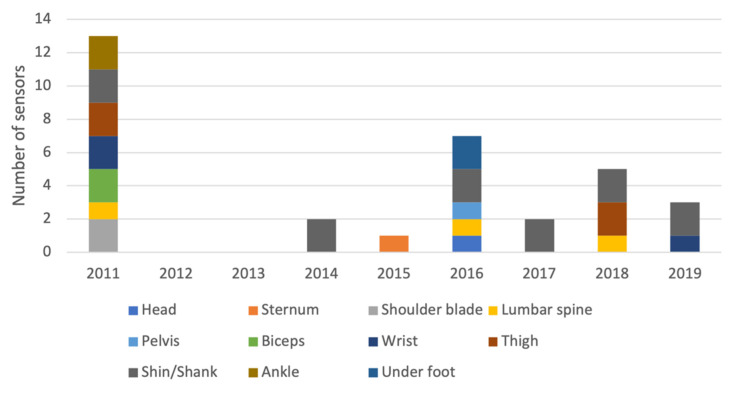
Number of sensors per body location for articles in Table 5 and Table 6, per publication year.

Continuing with the studies using classification methods/models (with or without machine learning algorithms (i.e., the articles in Table 5 and Table 6), Figure A3 in Appendix B) shows that most articles reported on sensors located on the lower body with the exception for the publication years 2011 and 2015. However, in 2011, 6/7 of the reported upper body sensors were used in one of the articles where a total of ten sensors were used [20], and only one article was included from 2015. The two articles published in 2014 [27] and 2017 [36] have the same main author and report on the use of sensors located at the shin/shank. The two included articles from 2016 use five different sensor locations, and 6/7 of the sensors were used in one article [29] where most of them were located on the lower body (shins/shanks and under the feet soles), and two of them on the upper body (head and pelvis). The other article from 2016 [32] used a sensor located on the upper body (lumbar spine). Only one article from 2018 [46] used sensors located both on the upper (lumbar spine) and lower body (thighs, and shins/shanks). In 2019, [51] used a sensor combining a 3D accelerometer and a photoelectric heart rate sensor located on the wrist, while the other study [49] positioned the sensors at the shins/shanks.

**Figure 7:**
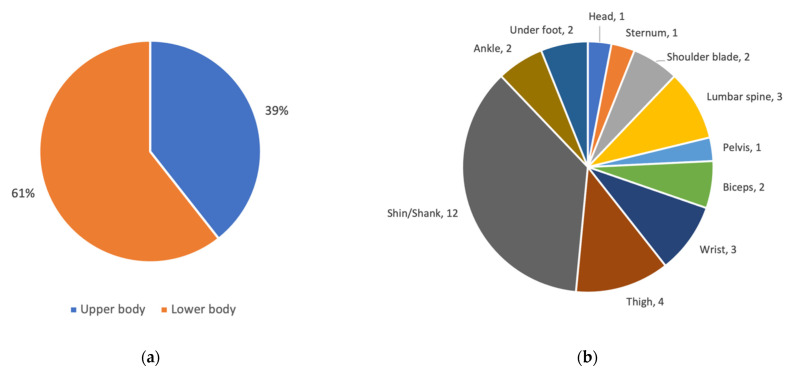
Information on sensor locations for articles in Table 5 and Table 6, i.e., the articles using classification methods/models with or without machine learning algorithms: (**a**) Distribution of sensors located at the upper or lower body; (**b**) Number of sensors per body location.

To summarize, 61% of the wearable sensors used in studies using classification methods/models (with or without machine learning (Figure 7a) were located on the lower body. Figure 7b shows that the most common body location on the lower body was the shin/shank (12 sensors). Other lower body locations used more than once include thigh (4), under foot (2), and ankle (2). Upper body locations used more than once include lumbar spine (3), wrist (3), biceps (2), and shoulder blade (2). Hence, the body locations used in studies using classification methods/models with or without machine learning, are quite different from the body locations used in the studies performing discrimination by features where 64% of the sensors were located on the upper body.

### 3.3. Signal Processing

The analysis of methods used for signal/data processing and analysis in the 33 studies identified that the used signal processing approaches could be classified according to three main categories: discrimination by feature selection (22 studies presented in Table 4), classification by use of classification methods/models with and without machine learning algorithms (5 studies without machine learning algorithms presented in Table 5, and 6 studies with machine learning algorithms presented in Table 6).

#### 3.3.1. Sensor Features

The number of sensor features selected for fall risk assessment analysis (either discrimination or classification) varied from one to hundreds between different studies.

Among studies discriminating by feature selection, i.e., performing statistical analysis directly on selected features (Table 4), most of the studies (14/21) used up to 10 (4–10) sensor features and four studies used 15–21 sensor features [21,34,41,47]. The highest number of sensor features used among the studies was 60 [31]. However, this number differed significantly from the other studies discriminating fall risk by feature selection. Some studies evaluated the discriminatory capabilities of generated sensor features, e.g., Step Stability Index (SSI) [25], Local Dynamic Stability (LDS) [30], and Biometric Signature Trajectory (BST) by [45].

In the studies using classification models without machine learning algorithms (Table 5), three articles from the same main author [27,36,49] used over 40 (44–71) sensor features in regularized discriminant classifier models. The other two studies [22,23], which both used regression models, utilized 10 and 14 sensor features respectively.

The studies using classification methods/models with machine learning algorithms (Table 6) used more sensor features than studies assessing fall risk based on feature extraction (Table 4) and on classification models without machine learning (Table 5). Two studies [29,46] used approximately 150 sensor features, while two other studies [20,32] used approximately 70 sensor features for four different machine learning algorithms. One study [28], which built on the machine learning classification algorithm decision tree (DT), used only seven sensor features. A total of 38 sensor features were combined with 210 variables in Resident Assessment Instrument—Home Care (RAI-HC) and analyzed using machine learning algorithms [51].

#### 3.3.2. Feature Selection

All studies employed feature selection, regardless of whether they used statistical analysis directly on the selected features to assess fall risk or used the selected features in classification methods/models which used machine learning algorithms or other types of classifiers.

Most studies used statistical tests in the feature selection process. Some articles stated that the Shapiro–Wilk test had been used to identify whether data was normally distributed prior to feature selection [43,47,51]. In studies where the data met requirements for parametric tests, those were used. Here, comparison tests including Analysis of variance (ANOVA) (10/33) and *t*-test (9/33) were mostly used. Analysis of co-variance (ANCOVA) was used together with ANOVA in [35] and combined with Multivariate ANCOVA (MANCOVA) in [40]. Moreover, correlation tests using Pearson’s correlation were employed in some studies [22,35,38,39,43]. When data did not fulfil requirements for parametric tests, non-parametric tests including Wilcoxon-signed-rank [19,25,34,41], Kruskall–Wallis tests [19], χ2 tests [24,50], Mann–Whitney test [22,25,34,41,42], Friedman test [34,43], Fisher’s exact test [41], and Tukey’s test [47] were used.

In Table 4, these feature selection methods were mostly used for assessment of individual difference and significance, i.e., which of the features to be included in fall discrimination analysis. However, in Table 5 and Table 6, the methods mostly tended to prepare data for classification models and machine learning algorithms, catering for the prediction and assessment of the classification ability or performance.

#### 3.3.3. Fall Risk Assessment

The 22 studies in Table 4 employed selection and comparison of features by performing statistical tests to discriminate between fallers and non-fallers or to classify individuals as fallers or non-fallers. The majority of these articles (17/22) compared the features between groups by performing different statistical tests with the aim of identifying features with significant discrimination ability. Some articles (5/22) proposed novel or valid measures from sensor measurements and analyzed the feasibility of those, i.e., a novel measure of SSI [25], LDS [30], refined composite multiscale entropy (RCME) and refined multiscale permutation entropies (RMPE) [31], BST [45], and Comprehensive Gait Assessment using Inertial sensor (C-GAITS) score [50].

In total, 11 studies employed classification methods/models with and without machine learning to discriminate between groups with different fall risk or to classify individuals as according to fall risk. Five of those studies (presented in Table 5) employed classification models without machine learning algorithms for fall risk assessment. Most commonly used models were regression models (4 studies) and discriminate classifier models (3). In addition, six studies (presented in Table 6) used machine learning classification algorithms, including adaptive boosting (Ada Boost) (1 study), boosted tree (BT) (1), bootstrap aggregation (bag) (1), DT (3), k-nearest neighbors’ classifier (KNN) (1), logistic regression (2), naïve Bayes (NB) (4), neural network (NN) (1), radial basis function network classifier (RBNC) (1), random forest (RF) (2), and support vector machine (SVM) (4) to assess fall risk

### 3.4. Identification of Triads and Assessment of Applicability on Classification Methods/Models

As described in Section 3.1.5, the current review reports on SFRA performed under supervised, semi-supervised, and unsupervised conditions (such as ADL or free-living gait) and the degree of supervision varied between article categories. For example, all studies using classification models without machine learning were supervised while only 50% of the studies using classification methods/models with machine learning algorithms were supervised.

A previous systematic review and meta-analysis of best available evidence of optimal combinations of sensor locations, tasks and feature for fall risk assessment [14] discussed discriminating sensor features of four certain tasks while wearing sensors. The six recommended triads were: (1) angular velocity—walking—chins, (2) frequency—walking—lower back, (3) frequency—walking—upper back, (4) linear acceleration—quiet standing—lower back, (4) linear acceleration—quiet standing—lower back, (5) linear acceleration—sit-to-stand/stand-to-sit—lower back, and (6) temporal—TUG—shins. The three not-recommended triads were: (1) angular velocity—walking—lower back, (2) frequency—walking—shins, and (3) linear acceleration—walking—shins [14]. In the current review, it was not possible to outline the aforementioned triads for the 11 studies performing classification methods/models, i.e., the studies in Table 5 and Table 6. The main reason for this is the fact that they present methods rather than sensor features. Further, the current review identified several assessment tasks that were not included in [14], for example reaction tests, stair ascent and decent, ADL, balance tests in different conditions, and an UEF test. Most of these newer tasks were used in studies performing discrimination by feature selection. Therefore, rather than trying to identify triads like the ones in [14] or counting sensor types/sensor locations for all studies, Section 3.2.1, Section 3.2.2 and Section 3.2.3 present information on number of sensors, sensor types, and sensor locations, differences between article categories as well as trends during 2011–2019. Nevertheless, an analysis relating to the previous systematic review by Montesinos et al.’s triads [14] has been conducted.

Starting with the studies performing discrimination by feature selection (i.e., the studies in Table 4), most sensors were located on the upper body and most of them were located on the lumbar spine. The lower back was included in the triads recommended by [14] for quiet standing and stand-to-sit/sit-to stand tasks but not TUG. For TUG, the recommended triad included temporal-shin. Only the studies [39,40] used TUG as an assessment task, [40] used the recommended sensor location, i.e., the shin. However, none of them presented results that distinguished fallers from non-fallers by using temporal sensor features. Four studies included standing balance tests at different conditions, VR included. Excluding the VR study [38], the three other studies [34,43,48] used sensors located on the lower back or pelvis. Hence, while not being assessment tasks listed in [14], the sensor location mimics the one in the recommended triad (4) above.

Regarding the walking task, [14] identified both recommended and not-recommended triads with respect to the lower back. Eight studies used different walking tasks. These included also walking in different conditions, daily life walking, walking in stairs, 6MWT and 15 m walking tests. The sensor was located on the lower back in seven studies, and on a nearby location (pelvis) in one study. However, the dimensionality of the collected sensor data varied. In the studies [19,24,25,31,41,42], features from one or more 3D accelerometers were used. A combination of 3D accelerometer and 3D gyroscope features was used in [50], and an even more complex combination (3D accelerometer, 3D gyroscope, 3D magnetometer, and barometer features) was used in [44]. Several of the studies using walking as the assessment task also used more than one sensor but the location of them varied. We note that the triad linear acceleration—walking—lower back was not identified as a recommended triad in [14].

The triad angular velocity—walking—lower back was identified as not-recommended in [14]. Nevertheless, gyroscope features were included in the C-GAITS score [50]. Finally, Table 4 includes two studies [21,30] including the sit-to-stand assessment task. Both used one or more sensors providing 3D accelerometer features. For sit-to-stand, the in recommended triad (4) above includes linear acceleration—lower back. This sensor location was used in [30] but the sensors were positioned on one of the thighs and sternum in [21].

The triads by [14] are not directly applicable for the studies using classification models/methods. The majority of the sensors used in the studies presented in Table 5 and Table 6 were located on the lower body with shin/shank being the most common location. Three of the studies in Table 5 [27,36,49] used TUG as the assessment task. All of them positioned the sensors on the shin/shank, i.e., the same location as in the recommended triad (6) above for TUG which included temporal-shins. One study, [22] was conducted by the same research group but used walking as an assessment task with sensors located on the shins/shanks. It should be acknowledged here that although the Shimmer sensor (i.e., a combination of 3D accelerometer and 3D gyroscope features) was used during the assessment, the research has resulted in a commercial quantitative TUG assessment tool called QTUG which is provided by the company Kinesis Health Technology.

One article [23] used both walking and TUG as assessment tasks. The location chosen for a 3D accelerometer was the lower back. Also, this triad was not identified by [14].

None of the recommended triads for walking (1–3 above) include linear acceleration, neither does the recommended triad for TUG (6 above). Hence, the studies in Table 5 show that it is possible to use also other triads when using classification models/methods. The assessment tasks vary significantly between the studies in Table 6, therefore, no further analysis on body locations and identification of triads is provided here.

### 3.5. Statistical Analyses on the Sensor-Based Methods’ Capabilities to Assess Fall Risk

Statistical analyses were performed on the SRFA methods’ capabilities to assess fall risk, either to discriminate between groups with different fall risk or to classify individuals as faller/non-faller. Methodological data and main findings on discriminatory capabilities of sensor features and classification methods/models are presented in Table 7 and Table 8. Methodological data and classification performance of sensor features, and classification methods/models are presented in Table 9 and Table 10.

#### 3.5.1. Capability in Discriminating Groups with Varied Level of Fall Risk

Eighteen (18/33) studies analyzed the SFRA methods’ capabilities to discriminate between groups of older adults with different fall risk, mostly fallers/non-fallers. Most of them (15/33, see Table 7) evaluated the discriminative capability of sensor features and a few (3/33, see Table 8) evaluated the discriminative capability of classification methods/models.

The 15 studies evaluating capabilities of sensor features to discriminate between groups (presented in Table 7) included 5–122 fallers (mean 31, median 20.5, SD 28). Only 2/15 studies identified fallers based on PRO data, one for 12 months and one for 6 months. Each study identified 1–6 sensor features which differed significantly between groups of participants with different fall risk levels. Almost half (7/15) of the studies identified sensor features related to gait, both more complex measures [25,50] and specific gait characteristic features such as within walk variability [35]. Moreover, one study identified that stair descent rate significantly differed between multiple- and non-multiple-fallers [41]. Five studies identified that features related to balance were significantly different between groups, for example during tandem stand [34], in regain of balance after movement initiation [26] and upon external stimuli [43].

The three studies evaluating the capabilities of classification models/algorithms to discriminate between groups (presented in Table 8) included 31-1637 fallers (mean 574, median 54, SD 752). One of the studies used an exceptionally large sample to validate a model that had been previously reported [49]. One of the studies [28] identified fallers based on PRO data, and a period of 12 months was used. Both classification methods/models with [28] and without [22,49] machine learning were evaluated. Two of the articles stated that model validation was performed.

#### 3.5.2. Capability in Classifying Individuals as Fallers/Non-Fallers (or Equivalent)

Fifteen (15/33) studies analyzed the SFRA methods’ performance in classifying older adults as fallers/non-fallers or equivalent. Seven of them evaluated classification performance of sensor features directly (Table 9) and eight evaluated classification methods/models which used sensor derived features (Table 10).

The seven studies evaluating performance of sensor features to classify individuals (presented in Table 9) included 16–50 fallers (mean 41, median 40, SD 14). Only two of them identified fallers based on PRO data, both for 12 months. Some studies analyzed features selected from sensor signals, e.g., gait speed [19] or HR derived from upper trunk accelerometry [24]. Others analyzed generated features, e.g., SSI [25], LDS [30], RCME, and RMPE [31].

**Figure 8:**
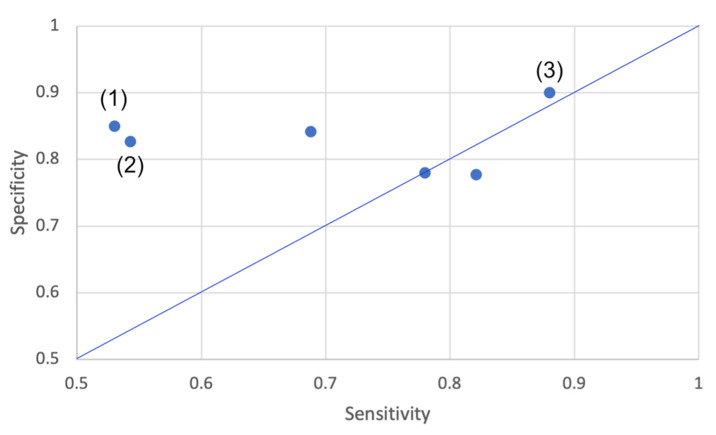
Specificity versus Sensitivity for studies reporting classification performance of sensor derived features. The blue line indicates specificity = sensitivity. (1), (2), and (3) denote values from [46], [44] and [37], respectively.

Six (6/7) studies reported Area Under Curve (Operating Characteristics-curve) (AUC)-values (range 0.67–0.90), half of them reported 95% confidence intervals (CIs) of the average AUCs and four of them reported AUC-values of 0.81 and higher. These six studies also reported values of sensitivity, i.e., the probability of classifying a true faller as faller, (range 53–88%) and specificity, i.e., the probability of classifying a true non-faller as non-faller (range 72–90%). As shown in Figure 8, 5/6 of these studies reported a specificity that was at least as high as the sensitivity. This indicates that the methods’ performance in classifying non-fallers was as least as high as their performance in classifying fallers. For example, ref [39,42] reported sensitivity values of 53% and 54.3%, respectively (see values marked with (1) and (2) in Figure 8). Ihlen et al. [31] reported the highest sensitivity (88%, see value marked with (3) in Figure 8).

Eight studies evaluated classification performance of sensor-based classification methods/models. One of them did not report on number of fallers in their sample but the other seven studies, presented in Table 10, included 11–33 fallers (mean 21, median 22, SD 7), all identified based on RE data.

Type of metrics used to report on classification performance varied between studies: most studies (7/8) reported classification accuracy with best performance values in the range 70–91%. In addition, values on sensitivity (best values of studies in range 36–100%), specificity (best value of studies in range 55–100%) and AUC-values (best value of studies in range 0.67–0.93) were reported. Three of the eight studies presented CIs for the reported values and three studies [23,42,48] reported performance metrics of clinical fall risk assessment methods for comparison.

More than 60% of the studies (5/8) evaluated the performance of models using machine learning algorithms. Each study evaluated the performance of three to six models such as NB, SVM, and multi-layer perceptron NN. Three of the four studies that included SVM-based models in their comparisons identified that this type of machine learning algorithm resulted in the best performance [29,32,46]. The remaining 40% of the studies (3/8) evaluated classification models based on logistic regression algorithms [23] and regularized discriminant classifier algorithms [27,36].

**Figure 9:**
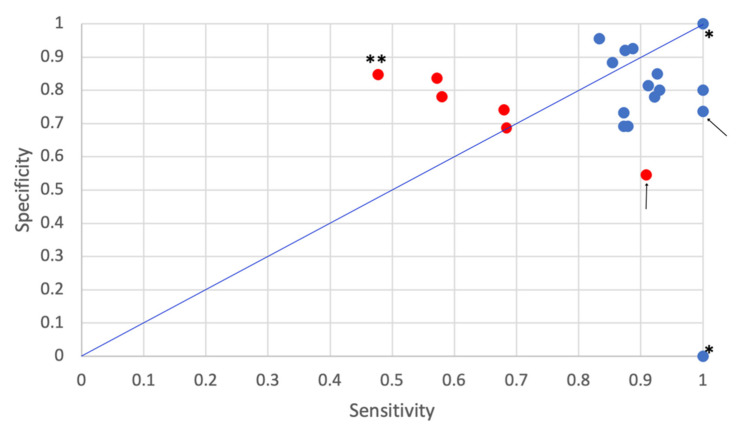
Specificity versus Sensitivity from studies using classification methods/models (presented in Table 10). Data from studies using models with machine learning are represented by blue dots and data from studies using models without machine learning are represented by red dots. The blue line indicates specificity = sensitivity. Data from evaluations using methods other than CV (hold-out or independent dataset) are marked with arrows. Two data points from [29] are marked with asterisks and one data point from [41] is marked with double asterisks.

Accuracy values reported from studies using classification models with machine learning (79.7–91%) were higher than accuracy values reported from studies that used classification models without machine learning (70–72.7%) (see Table 10). It should be noted though that only the highest achieved accuracy value per classification model is reported in this review. Moreover, studies employing models with machine learning reported higher sensitivity and specificity values compared to classification methods/models using other types of classifiers (see Figure 9). Here, the lowest sensitivity value, presented by [27], was below 50% (see data point marked with double asterisks in Figure 9). However, the study by Caby et al. [20], which compared four different machine learning algorithms, reported sensitivity values of 0 and 1 (see data points marked with one asterisk in Figure 9).

All studies validated their classification models, mostly by cross validation (CV). However, one study used a hold-out method with 75% of the data in the training set and 25% in the validation set [29], and one study used an independent dataset for model validation [36]. In both cases, the reported specificity was lower than the sensitivity (see data points marked with arrows in Figure 9), and this indicated that their performance in classifying fallers were at least as high as their performance in classifying non-fallers. Moreover, one of the studies which used CV reported that pruning was used in the model training to avoid overfitting [51].

## 4. Discussion

This article presents a systematic review of evaluations of SFRA methods in peer reviewed literature published 2010–2020. A total of 389 publications were screened for eligibility and 33 articles were included in the final assessment.

The current review identified that the most studied population was community-dwelling older adults. Although sample sizes varied widely between studies, 33% (12/35) of the samples in the 33 included studies had at least 100 participants. This percentage was higher than what had been identified in the review by Rucco et al. [15] which reported that only 10% (4/42) of their included studies had more than 100 participants. The review by Shany et al. [7] pointed out the need for high-quality validations of concepts that had been established as proof-of-concept in previous research. The current review identified one example of a large-scale validation published in 2019 [49] with a sample of 6295 participants.

RE data was identified as the most used comparator in the included studies. This result is in accordance with previous reviews which have identified RE data alone [14] or in combination with CLIN data [8,9,13] as the most common methods for generating outcomes to compare SFRAs. Although the need for using PRO data has been emphasized in previous reviews [7,8,9,13], and that a positive trend of increased use of PRO data was identified by Shany et al. [9], the current review could not identify an increase in prospective studies over the publication period.

The proportion of fallers (or equivalent outcomes indicating increased fall risk) in study samples varied between 14 and 71% with an average of 44%. The same range was reported in [15]. However, thresholds used to define a person with increased fall risk (at least one or two falls) varied. Most of the included studies based the SFRA on supervised assessment tasks. This is positive with regards to the need for research to support supervised SFRA and not only focus on unsupervised SFRA which was described in [7].

The number of different sensor types used in the included articles increased over time: while only 1–2 sensor types were used in the 10 articles published during 2011–2015, the number of sensor types increased from 2016. A higher number of sensor types was used among studies performing discrimination by feature selection than by those using classification methods/models. Moreover, the accelerometer, commonly used according to Bet et al. [16], was not used at all in the studies using classification methods/models that were published during 2017–2019.

This review identified that studies performing discrimination by feature selection and studies using classification methods/models differed in sensor locations used: while studies using classification methods/models mostly used sensors located on the lower body (shin/shank was the most common location), studies using feature selection mostly used sensors on the upper body (lumbar spine was the most common position). Another review by Bet et al. [16] also analyzed sensor locations and found that the most common location was the waist (8 articles), followed by the lumbar region (7), ankle (4), pelvis (4), and head (3). It is worth noticing here that different terminologies may possibly be used to denote the same sensor location. For example, [14] identified recommended and not-recommended triads including the shins but lists no articles including features from the ankle, while [16] identified four articles with sensors located on the ankle but none on the shin. Further, while [16] reported that the most frequently used locations were the waist and lower back (lumbar spine), [14] stated that the most common placement was the lower back (approximately L3). In addition, Rucco et al. [15] used the notation trunk for sensors located at L3, L5, sternum, waist, pelvis, neck, and chest. Hence, a direct comparison of results obtained in this review with results from the previous reviews is not straightforward.

The review by Sun and Sosnoff [13] focused on four major sensing technologies (inertial sensors, video/depth camera, pressure sensing platform and laser sensing) for SFRA in older adults. The authors presented outcome measures related to different assessment tasks (steady state walking, TUG test, standing postural sway, and dynamic tests) [13]. Howcroft et al.’s review [8] focused solely on inertial sensors. The current review included studies using wearable or mobile inertial sensors used to characterize movements by extracting features from sensor signals. Hence, the range of sensors used in the included studies was more limited in this review compared to the range reported in [13] but somewhat broader than the range reported in [8].

In accordance with the review by Shany et al. (2015) [9], selected features, methods for selecting/extracting them, as well as the number of features incorporated into each model varied substantially between studies. Shany et al. presented both numbers of features subjected to analysis and numbers of sensor features. In addition, they highlighted uncertainty of numbers by using the symbol “?” [9].

Montesinos et al. [14] identified strong/very strong associations between fall risk assessment outcomes and nine triads (combinations of a feature category, a task, and a sensor placement). In the current review, it was not possible to outline triads for the 11 studies performing fall risk assessment using classification methods/models. Further, several assessment tasks not included in Montesinos et al.’s [14] analysis were identified in the current review. Most of these newer tasks were used in studies performing discrimination by feature selection. Rather than trying to identify triads like the ones outlined by Montesinos et al. [14] or counting sensor types/sensor locations for all studies, the current review has instead presented information on sensor locations and sensor types used for studies performing discrimination by feature selection and classification methods/models, respectively.

Previous reviews have categorized SFRA signal processing methods differently compared to this review. For example, Howcroft et al. [8] claimed that regression models were employed to predict fall risk in 65% of their included studies. Other methods employed were mathematical classifiers (25%), DT (15%), NN (15%), SVM (10%), and cluster analysis (10%). Some of the studies (30%) employed more than one method. In addition, Sun and Sosnoff [13] presented a diverse collection of quantitative models/methods including logistic regression, linear regression, RBNC, SVM, NB, multi-layer perceptron NN, Locally Weighted Learning, DT, Cluster analysis, kNN, NN, neuro evolution of augmenting topologies (NEAT), and discriminate analysis employed to predict fall risk [13]. Both mentioned reviews categorized regression model as a classification method [8,13]. On the contrary, Bet et al. [16] used two main categories of data processing (feature extraction and machine learning techniques) in their analysis of included articles. They classified only data processing methods that carried out fall risk assessment by feature comparisons using statistical tests as “feature extraction” [16]. Notably, the current review categorized signal processing methods based on both the type of methods and on the results that the methods produced. In Table 4, seven articles employed logistic regression, i.e., logistic regression [41]; logistic regression and ROC curve [42]; logistic regression and ANOVA regression [37]; stepwise logistic regression [39]; stepwise logistic regression and ROC curve [19,24]; and univariate logistic regression [30]. In these articles, logistic regressions are used with the purpose to identify individual significant features associated with fall risk but not directly for classification. Therefore, these signal processing methods are categorized as “feature selection” in this review. Moreover, single linear regression, which was employed only in one article to assess the correlation between C-GAITS score and walking speed [50], was not characterized as a classification method. In Table 5, four articles employed logistic regression as a classification model [22,23,36,49]. These articles developed classification models based on regression models with related data. Moreover, in Table 6, a logistic regression model was employed as a machine learning classification model in one article by Yang et al. [51].

The included studies evaluated either the SFRA methods’ capabilities to discriminate groups of older adults with different fall risk (55% of the studies) or their performance in classifying individuals according to fall risk (45% of the studies). The SFRA methods were either using sensor features or classification methods/models (with and without machine learning) for discrimination/classification. This review identified a large number of sensor features (47% of them related to gait) and three classification models were identified to differ significantly between groups with different fall risk levels. Moreover, the review identified that classification performance was mainly reported using accuracy (highest values per feature/model 70–91%), sensitivity (highest values per feature/model 36–100%), specificity (highest values per feature/model 55–100%) and AUC (highest values per feature/model 0.67–0.93). The review by Sun and Sosnoff [13] presented data on full ranges of accuracy, sensitivity and specificity reported in their included articles while the current review only reported the highest values identified for each of the evaluated SFRA methods. Moreover, the review by Sun and Sosnoff [13] and the current review had only four studies [19,20,23,24] in common. Hence, Sun and Sosnoff [13] present lower values in the minima of ranges for accuracy, sensitivity and specificity than the current review. In general, the methods’ Specificity (performance in classifying non-fallers) was higher that their Sensitivity (performance in classifying fallers) in the current review. In accordance with the previously identified need to compare accuracies of SFRA methods with accuracy of CLIN data [8], the current review identified three studies [23,42,48] that reported performance metrics of clinical fall risk assessment methods for comparison.

Howcroft et al. [8] have previously pointed out that the reported accuracy values exceed the theoretical maximal accuracy (81%) for SFRA prediction of at least 1 fall in the upcoming year calculated by [10]) and concluded that prediction performance is over-estimated in current literature, mainly due to small samples, large feature pools, model overfitting, lack of validation, and misuse of modelling techniques. The current review identified one study which reported an idealistic model performance (Error = 0, Sensitivity = 1, Specificity = 1) [20] and five studies that reported model classification accuracy values exceeding 81% [27,29,32,46,51]. All these studies, except [27], used machine learning algorithms. The current review identified that all the studies presenting fall risk classification performance also reported on model validation methods. Although CV was used in most cases, one study performing validation with an independent sample [36] was also identified. This is a validation method that has been recommended by [9]. In addition, one example of the hold-out method was identified, data from 75 participants was included in a training set and data from 25 participants was used in a test set [29]. The identified use of model validation among the included studies in the current review is higher than the levels identified by Sun and Sosnoff [13]. Only 50% of their included studies had applied the recommended model validation techniques (including leave-one-out CV, ten-fold CV, 0.632 bootstrap technique and hold-out method).

## 5. Conclusions

This review identified evidence of SFRA, both in terms of discriminative capacity and classification performance:

(1) A large number of sensor features (almost 50% related to gait) and three classification (one with machine learning) models using sensor features (related to gait and stair descent) differed significantly between groups of older adults with different fall risk level.

(2) Six studies reported on sensor features (1–5 features per study, in one study combined with the Tinetti balance score) being able to classify individuals as fallers/non-fallers (or equivalent) with AUCs of at least 0.75. Five of these six studies used only 3D accelerometers and one used only gyroscope data. Assessment tasks monitored were walking (4/6 studies), TUG test (1/6), and standing balance (1/6).

(3) Seven studies reported on classification models (four with machine learning and three without) being able to classify individuals as fallers/non-fallers (or equivalent) with accuracies of at least 84% and/or AUCs of at least 0.74. All these studies used accelerometers, either alone (1 study) or in combination with 1–5 other sensors including gyroscopes (4 studies), magnetometers (1), pressure sensors (1) and heart rate sensor (1). The number of sensor features analyzed in these studies ranged between 38 and 155. Although more than half of the studies (4/7) used clinical tests (mainly TUG test) as assessment task, ADL (2 studies) and walking (1) were also used.

However, the review also identified several factors previously reported to increase risk of bias [7,8,9,12,13,14,15,16]:

(1) The use of prospective study design was limited among the included studies and no positive trend over the publication period could be identified. Two thirds of the included studies used cross-sectional design with RE and/or CLIN data as outcomes to compare SFRA with. Potential sources of biases associated with RE data include limited accuracy of recall of falls in the elderly [55] and risk of altered motion patterns due to history of falls [9]. Moreover, clinical assessments can introduce study bias since they are often assessed subjectively and do not achieve 100% clinical accuracies [7,13].

(2) Approximately one third (12/35) of the samples in the included studies had at least 100 participants. Although this proportion was higher than the 10% identified in [15], this indicated that most of the included samples were limited. Inadequate sample size has been identified as an important risk of bias in SFRA research [9]. Moreover, 14–71% (44% in average) of the participants in the included samples had elevated fall risk (i.e., were labelled as fallers, frail, at risk, etc.), the same range was reported in [15]. Insufficient raw numbers of events can contribute to small-sample bias [56] and samples with very few fall events relative to the total sample size can lead to distorted models [9,57]. Our comparison of sensitivity and specificity identified that the SFRA methods’ performance in classifying non-fallers (or persons not at risk) was at least as high as their performance in classifying fallers (or percentage). This might be a consequence of low in-sample proportions of persons with elevated fall risk.

(3) Low consensus was identified among the included studies in number and type of sensor features used in SFRA. Moreover, the number of features was highest among studies using classification models with machine learning. This result illustrated that the previously highlighted “curse of dimensionality” remains a challenge in the model selection process of SFRA which might contribute to sample bias [9].

(4) Although all included studies using classification models performed model validation, the most common validation method was CV which has been identified to increase the risk of bias in estimated performance [9], especially for small data sets [58]. However, this review also identified model validation using independent datasets [23] and hold-out technique [29] which, according to [9], are preferred over CV.

Hence, future SFRA research should continue to reduce risk of bias by further implementing methodological improvements.

## 6. Limitations

The search terms used, specifically the required combination of “risk” and “assessment” may have limited the search results of this review. However, the number of articles included in the current review (*n* = 33) is in the same range as previous reviews (*n* = 22 in [13], *n* = 40 in [8], *n* = 24 in [9], *n* = 13 in [14], *n* = 42 in [15] and *n* = 29 in [16]). Interestingly, only 9/33 of the included studies [19,20,21,22,23,24,25,27,41] overlapped with previous reviews.

## Figures and Tables

**Table 1:** Review elements.

Objective/Aim	Inclusion Criteria	Exclusion Criteria
To make a systematic review of the performance of wearable sensor systems in discriminating/classifying older adults according to their fall risk. The following PICO elements [18] were used: **Population of interest:** Persons being 60 years and older without severe cognitive impairment. **Investigated test result:** Person classified as faller according to SFRA **Comparator test result:** Person classified as non-faller according to SFRA **Outcome:** Person classified as faller (or equivalent, e.g., high risk) according to Retrospective falls history (RE), Prospective fall occurrence (PRO) or Clinical assessment methods (CLIN) **Study design:** Prospective and Cross-Sectional studies	1. Original, peer-reviewed journal articles/conference papers published and indexed during Jan 2010–April 2020 in English language. 2. Participants labelled as (single/multiple-) fallers/non-fallers or high/low risk based on: RE data PRO data CLIN data A combination of a–c 3. Sample: N ≥ 10, age ≥ 60 years 4. Wearable or mobile inertial sensors used to characterize movements by extracting features from sensor signals. 5. Evidence of SFRAs in terms of (a) discriminative capacity (statistically significant discriminatory features) and/or (b) classification performance (accuracy, sensitivity, specificity). Inclusion criteria 2–4 were based on a previous systematic review of SFRA [14].	Papers must not include participants with severe cognitive impairment, e.g., dementia. Papers must not only include measurements of total physical activity by activity monitors.
		**Outcomes**
		(a) Qualitative data on features with statistically significant discriminative capacity (*p* < 0.05). (b) Quantitative data on classification performance (accuracy, sensitivity, specificity).

**Table 2:** An overview of databases, search phrases used during article retrieval, and information on search dates. The numbers indicate the number of identified articles in each search.

Database	Search Phrase	Search Date	Number of Articles
Web of Science Core Collection	TOPIC: (fall AND risk AND assessment) AND TOPIC:(inertial sensors) AND TOPIC:(geriatric OR older OR senior) AND TOPIC: (clinical)	20 September 2019	31
IEEE Xplore	TOPIC: (fall AND risk AND assessment) AND TOPIC:(inertial sensors) AND TOPIC:(geriatric OR older OR senior) AND TOPIC: (clinical)	30 October 2019	21
Web of Science Core Collection	TOPIC: ((fall AND risk AND assessment) AND (inertial sensors OR wearable OR technology OR device OR accelerometer OR gyroscope OR magnetometer) AND (geriatric OR older OR senior OR old) AND (clinical))	6 March 2020	129
IEEE Xplore	TOPIC: fall AND risk AND assessment AND clinical AND (“inertial sensors” OR wearable OR technology OR device OR accelerometer OR gyroscope OR magnetometer) AND (geriatric OR older OR old OR senior) Filters Applied: Conferences Journals	12 March 2020	32
Pubmed	((((fall[All Fields] AND (“risk”[MeSH Terms] OR “risk”[All Fields])) AND (“Assessment”[Journal] OR “assessment”[All Fields])) AND clinical[All Fields]) AND (geriatric[All Fields] OR older[All Fields] OR old[All Fields] OR senior[All Fields])) AND (“inertial sensors”[All Fields] OR wearable[All Fields] OR (“technology”[MeSH Terms] OR “technology”[All Fields]) OR (“equipment and supplies”[MeSH Terms] OR (“equipment”[All Fields] AND “supplies”[All Fields]) OR “equipment and supplies”[All Fields] OR “device”[All Fields]) OR accelerometer[All Fields] OR gyroscope[All Fields] OR magnetometer[All Fields])	27 March 2020	120
Medline	fall AND risk AND assessment AND clinical AND (“inertial sensors” OR wearable OR technology OR device OR accelerometer OR gyroscope OR magnetometer) AND (geriatric OR older OR old OR senior) Expanders Limiters: Apply equivalent subjects Journals: Scholarly (Peer Reviewed) Journals Source Types: Academic Journals Language: English	3 April 2020	281

**Table 3:** Characteristics of included studies. ADL = activity in daily life, FoF = Fear of Falling, FTSS = 5 times Sit-to-stand test, SEMI-SUP = Semi-supervised, SUP = Supervised, 6MWT = Six-Minutes Walking Test, UEF = Upper extremities’ function, UNSUP = Unsupervised, Val = Validation, VR = Virtual reality.

First Author, Year	Ref No.	Study Population	Faller/Non-Faller Labelling Method	Classification Outcome	Participants (% Fallers)	Assessment Task	Degree of Supervision
Bautmans, 2011	[19]	Other	Retrospective falls (6 months) and Clinical assessment (Tinetti, TUG)	*non* *-faller/faller*	81 (49%)	Walking (2 × 18 m)	SUP
Caby, 2011	[20]	Hospitalized patients	Retrospective falls (12 months) and Clinical assessment (Tinetti, Mini Motor Test)	*non-faller not at risk/faller at risk of falling*	20 (75%)	Walking (25m)	SUP
Doheny, 2011	[21]	Community-dwelling	Retrospective falls (60 months) and Clinical assessment (FoF and Cardiovascular risk factors)	*non-faller/faller (≥2 falls/1 fall seeking medical care*)	39 (49%)	FTSS	SUP
Greene, 2011	[22]	Community-dwelling	Retrospective falls (60 months)	*non-faller/faller*	114 (47%)	Walking (30 m continuous)	SUP
Marschollek, 2011	[23]	Patients, geriatric	Prospective falls (12 months)	*non-faller/faller*	46 (41%)	TUG test Walking (20 m)	SUP
Doi, 2013	[24]	Community-dwelling	Prospective falls (12 months)	*non-faller/faller*	73 (22%)	Walking (15 m)	SUP
Cui, 2014	[25]	Community-dwelling	Retrospective falls (12 months)	*non-faller/faller* (≥2 falls)	81 (49%)	Walking (3 conditions)	SUP
Ejupi, 2014	[26]	Community-dwelling	Retrospective falls (12 months)	*non-faller/faller*	104 (35%)	Choice Stepping Reaction Test	SUP
Greene, 2014	[27]	Community-dwelling	Retrospective falls (60 months) and Prospective falls (24 months)	*non-faller/faller*	RE: 909 (NA) PRO: 259 (NA) Val: 55 (0%)	TUG test	SUP
Brodie, 2015	[28]	Community-dwelling	Prospective falls (12 months)	*non-faller/faller/multi-faller*	52 (42% single, 17% multi)	Stair ascent	SEMI-SUP at research facility
Howcroft, 2016	[29]	Other	Retrospective falls (6 months)	*non-faller/faller*	100 (24%)	Walking (7.62 m, single- and dual-task condition)	SUP
Ihlen, 2016	[30]	Community-dwelling	Retrospective falls (12 months)	*non-faller/faller* (≥2 falls)	71 (45%)	4 FTSS at home for 1 day	SUP at home
Ihlen, 2016	[31]	Community-dwelling	Retrospective falls (12 months)	*non-faller/faller* (≥2 falls)	71 (45%)	Walking (daily life)	UNSUP 3 days at home
Iluz, 2016	[32]	Other	Retrospective falls (12 months)	*non-faller/faller* (≥2 falls)	71 (46%)	ADL (identified sit-to-walk and walk-to-sit transitions)	UNSUP 3 days at home
Mancini, 2016	[33]	Other	Retrospective falls (12 months) and Prospective falls (6 months)	*non-faller/faller/multiple-faller (RE)* *non-faller (PRO)*	35 (RE: 34% single, 20% multi; PRO: 20%)	ADL	UNSUP 1 week at home
Alqahtani, 2017	[34]	In residential care	Retrospective falls (12 months)	*non-faller/faller*	29 (66%)	Standing balance test	SUP
Brodie, 2017	[35]	Community-dwelling	Retrospective falls (12 months)	*non-faller/faller*	96 (34%)	ADL	UNSUP, 1 week at home
Greene, 2017	[36]	Community-dwelling	Retrospective falls (12 months)	*non-faller/faller*	22 (50%)	TUG test	SUP
Joseph, 2017	[37]	Patients, bedbound in hospital	Clinical assessment (UEF index)	*frail/non-frail*	101 (52%)	UEF assessment	SUP
Saldana, 2017	[38]	In continuing-care retirement community	Clinical assessment (Questions including retrospective falls and FoF)	*low-risk/at-risk*	13 (38%)	Standing balance with VR room rotation	SUP
Sample, 2017	[39]	Other	Retrospective falls (12 months)	*non-faller/faller*	148 (40%)	TUG test	SUP
Smith, 2017	[40]	Community-dwelling	Retrospective falls (12 months)	*non-faller/faller*	37 (43%)	TUG test (single-, motor- and cognitive dual task)	SUP
Wang, 2017	[41]	Community-dwelling	Prospective falls (12 months)	*multiple-faller/non-multiple-faller*	81 (14%)	Walking (flat surface and stairs)	SEMI-SUP at research facility
Bizovska, 2018	[42]	Other	Prospective falls (12 months)	*non-faller/faller/multiple-faller*	131 (27%, multi 11%)	Walking (25 m)	SUP
Ehsani, 2018	[43]	Other	Clinical assessment (Questions including retrospective falls, 12 months)	*high fall-risk/low fall-risk*	20 (50%)	Upright standing balance (eyes open/closed, with/without vibration)	SUP
Genovese, 2018	[44]	Patients	Determined by clinical partners	*high fall-risk/* *age matched low fall-risk*	50 (50%)	6MWT	SUP
Ghahramani, 2018	[45]	Community-dwelling	Retrospective falls (time period not presented)	*non-faller/multiple-faller*	45 (49% multi)	Turning test	SUP
Qiu, 2018	[46]	Community-dwelling females	Retrospective falls (60 months)	*non-faller/faller*	196 (42%)	Battery of clinical tests including assessment of standing balance, stability, sit-stand-transitions, walking, motor function, reaction and FoF	SUP
Del Din, 2019	[47]	Other	Retrospective falls (6 months (≥ 2 falls to identify fallers) and 18 months (0 falls to identify non-faller))	*non-faller/faller*	172 (71%)	Free living gait	UNSUP, 1 week at home
Ghahramani, 2019	[48]	Community-dwelling	Retrospective falls (12 months)	*non-faller/faller/multiple-faller*	86 (21% single, 44% multi)	Standing balance tests	SUP
Greene, 2019	[49]	Stratified sample	Retrospective falls (12 months)	*non-faller/faller*	6295 (14% single, 12% multi)	TUG test	SUP
Misu, 2019	[50]	Community-dwelling	Retrospective falls (12 months)	RE-12 *non-faller/faller*	378 (17%)	Walking (15 m)	SUP
Yang, 2019	[51]	Community-dwelling	Retrospective falls (3 months)	*non-faller/faller/multiple-faller*	40 (20% single, 40% multi)	ADL	UNSUP, 1 week at home

**Table 4:** Characteristics of SFRA methods which performed discrimination by feature selection. accel = accelerometer, AP = anterior-posterior, DTW = Dynamic Time Warping, EM = Expectation Maximization, GMM = Gaussian Mixture Models, gyro = gyroscope, HR = harmonic ratio, IMF = Intrinsic Mode Function, ML = medio-lateral, MML = Minimum Message Length, PLS-DA = Partial least square discriminatory analysis, RMS = root mean square, ROC = receiver operating characteristic, SEF = Spectral Edge Frequency, VRHMD = VR head-mounted display, VT = vertical.

Ref No.	Assessment Task	No. and Type of Wearable Sensor(s)	Sensor Position(s)	No. of Sensor Features	Feature Selection Methods	Wearable Sensor Features Able to Discriminate Significantly between Fallers/Non-Fallers
[19]	2 × 18 m walking	1 3D accel	Pelvis (sacrum between the spinae ilaca posterior superior)	6	ANOVA and *t*-test, Wilcoxon-signed-rank, Kruskall–Wallis tests, stepwise logistic regression with forward likelihood ratio and ROC curve	Gait speed
[21]	FTSS	2 inertial sensors (3D accel data used)	Anterior of right thigh, sternum	19	ANOVA and one-way ANOVA	Mean sit-stand-time, total jerk, total SEF, Mean sit-stand-sit SEF, Mean-stand-sit SEF, Mean sit-stand SEF
[24]	15 m walking	2 3D accel	Upper trunk (C7 spinous process) and lower trunk (L3 spinous process)	6	Independent *t*-tests or χ2 tests, stepwise logistic regression with forward stepwise selection and ROC curve	HR in VT direction in Upper trunk
[25]	Walking under 3 different conditions	1 inertial sensor (3D accel data used)	Lower back (belt)	1 ^1^	Wilcoxon Signed Rank and Mann Whitney test	SSI
[26]	Choice stepping Reaction Test (in exergame)	1 3D accel	Around neck (sternum height) inside clothes	6	Two-sided Student’s *t*-test	Reaction time, Total stability time
[30]	4 FTSS at home for 1 day	1 inertial sensor (3D accel data used)	Lower back (belt around waist)	1 ^2^	Univariate logistic regression and stepwise multivariate logistic regression with stepwise backward feature selection	LDS calculated by the Ihlen algorithm (Equation (2)) [52] with optimal identified parameter setting
[31]	Daily life walking	1 inertial sensor (3D accel data used)	Lower back (belt)	60	PLS-DA with a backward feature selection	RCME and RMPE for trunk acceleration and trunk velocity
[33]	ADL	3 inertial sensors (3D gyro data used)	Posterior trunk at about L5 (belt) and on the top of each foot (on shoes)	6	One-way ANOVA	RE-12: turn duration, mean peak speed of turning, mean number of steps/turns, Coefficient of Variation of turn angle; PRO-6: Coefficient of Variation of steps per turn
[34]	Standing balance test	1 2D accel and 1D load cell	Pelvis (Iliac crest)	16	Friedman test, Wilcoxon signed ranks test, Spearman rank correlation and Mann-Whitney U test	RMS sway acceleration in ML direction during semi-tandem stance
[35]	ADL	1 3D accel + barometer	Pending around neck	7	ANOVA, ANCOVA, Pearson’s correlation and Partial Pearson’s correction	Gait endurance and within walk variability in daily life
[37]	UEF assessment	2 3D gyro	Upper arm (near the biceps and to wrist)	8	ANOVA, Logistic regression (for nominal health outcomes) and ANOVA regression (for continuous health outcomes)	UEF index including speed, power and speed reduction
[38]	Standing balance with VR room rotation	1 VRHMD (6D inertial sensor + camera) and a force plate	Head	10 under each condition	One-sample *t*-test, signed rank test, paired *t*-test and Pearson’s correlation	VRHMD AP velocity while eyes open in VR module “balance”
[39]	TUG test	4 inertial sensor (3D accel and 3D gyro) and a force plate	Chest, lower back, each foot	8 (plus 9 post-urography parameters)	Stepwise logistic regression and Pearson correlation	Combination of Sit-to-Stand Duration, Stand-to-Sit Duration, Turn Peak Velocity, AP Sway Range, Height
[40]	TUG test under single- motor- and cognitive dual task	2 inertial sensors (3D accel and 3D gyro)	Anterior of shank (shin)	10	MANCOVA and ANCOVA	Cadence, stride velocity, stride time
[41]	Walking on flat surface and stairs	2 inertial sensors (3D accel data used)	Lower back and right ankle	15	Two-sample *t*-test, Fisher’s exact test, Mann-Whitney-U test, Wilcoxon’s signed-rank test, Benjamini-Hochberg adjustments, and logistic regression	Stair descent rate
[42]	25 m walking	3 3D accel	Trunk (near L5) and on both shanks (15 cm above malleolus)	6 (plus 3 Tinetti scores)	Mann-Whitney U test, logistic regression, and ROC curve	Only combined with Tinetti balance- and Tinetti total score ML trunk short term Lyapunov exponent was able to predict falls
[43]	Upright standing balance (eyes open/closed, with/without vibration	2 3D gyro	Lower back and shin	8	ANOVA, Friedman test, linear Pearson correlations	Local-control_slope_, for eye-closed when vibration stimuli were applied
[44]	6MWT	1 3D accel, 3D gyro, 3D magneto- and barometer	Lower trunk (L3 spinous process)	8	*t*-test	Walked distance, cadence, RMS of vertical acceleration, stride time
[45]	Turning test	4 inertial sensors (3D gyro used)	Chest, pelvis, and upper legs	1 ^3^	ANOVA, DTW algorithm	BST
[47]	Free living gait	1 3D accel	Lower back	21	Shapiro–Wilk test, Levene’s Test of Equality of Variances, general linear modelling, and Tukey’s test	Step velocity variability
[48]	Standing balance test	1 inertial sensor (3D gyro data used)	Lower back (above pelvis)	4	GMM, EM, and MML algorithm, ANOVA and ROC-curve	Standing with feet together sway index, standing with one foot in front sway index
[50]	15 m walking	2 inertial sensors (3D accel and 3D gyro)	Right heel (posterior surface) and trunk (L3 spinous process)	10	Unpaired *t*-tests/χ2 tests, unweighted least squares as extraction method and Cronbach’s alpha coefficient	C-GAITS score

^1^ Calculated from 12 IMFs where 8 IMFs from accel signals. ^2^ Generated from 4 different algorithms using optimal setting of lag size and sensor data. ^3^ Generated based on 4 sensor features.

**Table 5:** Characteristics of fall risk assessment systems using classification models without machine learning algorithms. CV = cross-validation, MGC = Minimum ground clearance, SagAngVel = Angular velocity in the sagittal plane.

Ref No.	Assessment Task	No. and Type of Wearable Sensor(S)	Sensor Position(s)	No. of Sensor Features	Feature Selection Methods	Models Able to Discriminate Significantly between Fallers/Non-Fallers
[22]	30 m continuous walk	2 inertial sensors (3D accel and 3D gyro)	Mid-point of anterior shank	10	Mann–Whitney Wilcoxon rank sum and Pearson’s correlation	MGC estimation by regression models (MGC model and MGC variance model) using the features mean SagAngVel at mid-swing points, mean absolute valued SagAngVel and min SagAngVel
[23]	TUG test and 20 m walk	1 3D accel	Lower back (belt around waist)	14	Wrapper feature selection algorithm (wrapper subset evaluator employing the simple logistic algorithm)	Logistic regression models CONV (using conventional clinical assessment data) and SENSOR (using sensor data from TUG and overall physical activity)
[27]	TUG test	2 inertial sensors (3D accel and 3D gyro)	Anterior of each shin, shank bone (tibial bone)	52	Sequential forward feature selection	Regularized discriminant classifier models using 52 temporal, spatial, turning, and rotational features from TUG
[36]	TUG test	2 inertial sensors (3D accel and 3D gyro)	Mid-point of left and right anterior shank (shin)	44	Nested CV	Classification model FRE_combined_ which combines FRE_sensor_ (regularized discriminant model using QTUG parameters during standing walking and turning) and FRE_clin_ (logistic regression model using clinical data on fall risk factors
[49]	TUG test	2 inertial sensors (3D accel and 3D gyro)	Mid-point of left and right anterior shank (shin)	71	One-way ANOVA	FRE_combined_ (i.e, the weighted average of the two FRE models FRE_sensor_ (regularized discriminant model using IMU-data from TUG + anthropomorphic data) and FRE_clinical_ (logistic regression model using clinical questionnaire data) Each mobility score (speed, turn, transfers, symmetry, variability) in mobility impairment score was significantly associated with falls history

**Table 6:** Characteristics of SFRA methods using classification methods/models with machine learning algorithms. F1-score = harmonic mean of precision and Sens, MCC = Matthew’s Correlation Coefficient, POM = Proportional odds models.

Ref No.	Assessment Task	No. and Type of Wearable Sensor(s)	Sensor Position(s)	No. of Sensor Features	Feature Selection Methods	Methods/Models Able to Discriminate Significantly between Fallers/Non-Fallers
[20]	25 m walking	10 3D accel	Mid-point of anterior shank	10	*t*-test using Holm correction, Behrens-Fisher test, forward wrapper selection algorithm family	RBNC, SVM, KNN, NB
[28]	Stair ascent	1 3D accel + 1 barometer	Lower back (belt around waist)	14	Spearman’s rank correlations, Kruskal–Wallis	Wavelet DT with adaptive threshold
[29]	Walking 7.62m under single and dual tasks	4 3D accel + 2 pressure sensing insoles	Anterior of each shin, shank bone (tibial bone)	146	Acc, F1-score, MCC	SVM, NN, NB
[32]	Identified sit-to-walk and walk-to-sit transitions in ADL	1 inertial sensor (3D accel data used)	Low back (belt)	72	4 machine learning algorithms in Matlab), linear regression analysis	Ada Boost, SVM, bag, NB
[46]	Battery of 5 clinical tests including assessment of standing balance, stability, sit-stand-transitions, walking, motor function, reaction, and FoF	5 inertial sensors (3D accel, 3D gyro and 3D magnetometer)	Low back, upper and lower legs	155	Two-sample *t*-tests, ROC analysis	Logistic regression, NB, DT, RF, BT, SVM
[51]	ADL	1 3D accel + 1 photoelectric heart rate sensor	Wrist	38 ^1^	One-way and two-way ANOVA, Kruskal–Wallis H test, multicollinear test, recursive feature algorithm in Caret R package	Three-class classification: POM and two machine learning algorithms (DT and RF) Binary classification: Three machine learning algorithms (logistic regression, DT, RF)

^1^ Combined with 210 variables in RAI-HC.

**Table 7:** Statistical analyses on the sensor-based features’ abilities to discriminate groups with distinct levels of fall risk. BMI = Body Mass Index.

Ref No.	No. of Fallers/No. of Participants (Faller/Non-Faller Labelling Method)	Sensor Features’ Performance in Discriminating Groups with Different Level of Fall Risk (Fallers/Non-Fallers)	No. of Features and Type of Assessment Task Able to Discriminate Groups with Different Level of Fall Risk (Fallers/Non-Fallers)
[21]	19/39 (RE-60, CLIN)	Fallers took significantly longer time to complete sit-stand transitions than non-fallers; Fallers exhibited increased jerk over the complete assessment than non-fallers; SEF was significantly higher for fallers than non-fallers for the total test, sit-stand-sit components, sit-stand and stand-sit transitions	6, sit-stand and stand-sit transitions
[25]	39/81 (RE-12)	The SSI was significantly higher for fallers than non-fallers under all three walking conditions (baseline with and without harness, obstacle negotiation with harness)	3, gait
[26]	36/104 (RE-12)	Significantly longer times to regain balance after movement initiation and slower stability time for fallers than for non-fallers.	2, stability/balance
[33]	19/35 (RE-12) 7/35 (PRO-6)	RE-12: Mean turn duration, mean peek speed of turning and mean number of steps/turn and the coefficient of variance of the turn angle were significantly different between multiple-fallers and non-fallers. Multiple-fallers had a longer turn duration, slower mean peak speed of turning, a higher number of steps/turn, and showed a lower coefficient of variance of turn angle than non-fallers. Multiple-fallers took a significantly higher number of steps/turn and showed a lower coefficient of variance of turn angle than fallers. PRO-6: 7/35 fell during the 6-month period. The coefficient of variance of steps per turn was significantly larger for fallers and multiple-fallers than for non-fallers.	RE-12: 4, turning and gait PRO-6: 1, gait
[34]	19/29 (RE-12)	RMS sway acceleration for ML direction during semi-tandem stance was significantly higher among fallers than non-fallers.	1, stability/balance
[35]	33/96 (RE-12)	After adjusting for demographics, fallers had significantly lower gait endurance and higher within walk variability in daily life than non-fallers.	2, gait
[37]	53/101 (UEF I)	The UEF index (adjusted for age, gender, BMI, age, discharge disposition) was a predictor for 30-day prospective falls. The UEF index, which assesses frailty, was higher in the frail group than in the non-frail group.	1, upper extremity
[38]	5/13 (CLIN incl. RE)	People at-risk of falling changed their head tilt in the AP direction significantly faster than people not at risk. Only reliable variables (identified from test-retest reliability evaluations) were included in the analysis.	1, stability/balance
[40]	16/37 (RE-12)	Fallers had a significantly higher cadence, higher stride velocity and shorter stride time than non-fallers.	3, gait
[41]	11/81 (PRO-12)	Stair descent rate was significantly higher among multiple-fallers than non-multiple-fallers	1, stair negotiation
[43]	10/20 (CLIN incl. RE)	When vibration was induced in the eyes-closed condition, the people with a high fall risk changed the local-control_slope_ significantly less than people with a low fall risk	1, stability/balance
[44]	25/50 (determined by clinical partners)	People with a high fall-risk had a significantly shorter walked distance, lower cadence, lower RMS (vertical acceleration) and higher stride time than people with a low fall-risk.	4, gait
[45]	22/45 (RE)	The DTW difference between the reference BST and each participant’s BST was significantly higher among elderly multiple fallers than non-fallers.	1, balance/stability
[47]	122/172 (RE-18/RE-6)	Step velocity variability was significantly lower among older adult fallers than older adult non-fallers.	1, gait
[50]	65/378 (RE-18)	Statistically significant lower C-GAITS score among fallers than non-fallers.	1, gait:

**Table 8:** Statistical analyses on the sensor-based methods’/models’ abilities to discriminate groups with distinct levels of fall risk. FRE = Fall Risk Estimate.

Ref No.	No. of Fallers/No. of Participants (Faller/Non-Faller Labelling Method)	Classification Models/Algorithms Included in Discrimination Method	Model Validation Method	Methods’/Models’ Performance in Discriminating Groups with Different Level of Fall Risk (Fallers/Non-Fallers)	No. of Features and Type of Assessment Task Able to Discriminate Groups with Different Level of Fall Risk (Fallers/Non-Fallers)
[22]	54/114 (RE-60)	2 regression algorithms of minimum ground clearance	NA	Fallers had a significantly lower mean SagAngVel at mid-swing points, mean SagAngVel absolute value and min SagAngVel than non-fallers.	3, gait
[28]	31/52 (PRO-12)	Machine learning algorithms (wavelet DT with adaptive threshold) using barometer and Accel features for classifying stair negotiation	Annotated video and 4-fold CV (x4 times)	PRO significantly correlated with reduced stair ascent stability (HR-AP, r = −0.35).	1, stair negotiation
[49]	1637/6295 (RE-12)	Regularized discriminant model (sensor data), logistic regression model (clinical data)	Validation of models previously reported	FRE_combined_ significantly associated with RE (F = 214.19, ρ < 0.0001). Each mobility score (speed, turn, transfers, symmetry, variability) in mobility impairment score was significantly associated with falls history.	NA

**Table 9:** Statistical analyses on the sensor-based features’ performance in classifying older individuals according to their risk of falling. Acc = Accuracy, BBS = Bergs Balance Scale, Err = Error, Sens = Sensitivity, Spec = Specificity.

Ref No.	No. of Fallers/No. of Participants (Faller/Non-Faller Labelling Method)	Sensor Features’ Performance in Discriminating Groups with Different Level of Fall Risk (Fallers/Non-Fallers)	Comment
[19]	40/81 (RE-6, CLIN)	Gait speed (cut-off 1.158 m/s): Acc = 77%; Sens = 78%; Spec = 78%; AUC = 0.83	
[24]	16/71 (PRO-12)	UT HR-VT: AUC = 0.81 (95% CI: 0.69–0.83; *p* < 0.001). Sens = 68.8% and Spec = 84.2% at cutoff value 1.89 based on the Youden index.	95% CI for AUC presented (as suggested in previous reviews)
[30]	32/71 (RE-12)	Kantz’ algorithm [53] with the best performing parameter: AUC = 0.73 (95% CI:0.60–0.85; *p* = 0.003) Ihlen’s algorithm [52] with the best performing parameter: AUC = 0.75 (CI:0.60–0.82; *p* > 0.001) Rosenstein’s algorithm [54] with the best performing parameter: AUC = 0.59 (CI:0.44–0.71; non-significant)	95% CI for AUC of as well as pairwise comparison of AUC of models presented (as suggested in previous reviews)
[31]	32/71 (RE-12)	Mean (Equation (1)) RCME for trunk acceleration: Sens = 0.84; Spec = 0.85; AUC = 0.81; Err = 0.15 Mean (Equation 1) RCME for trunk velocity: Sens = 0.78; Spec = 0.90; AUC = 0.83; Err = 0.15 Mean (Equation (1)) RPME for trunk acceleration: Sens = 0.88; Spec = 0.90; AUC = 0.88; Err = 0.11 Mean (Equation (1)) RPME for trunk velocity: Sens = 0.75; Spec = 0.87; AUC = 0.82; Err = 0.18	
[39]	59/148 (RE-12)	Height, sit-to-stand duration, stand-to-sit duration, turn peak velocity, AP sway range: Sens = 54.3%, Spec = 82.7%; max re-scaled R2 = 0.3244	
[42]	50/131 (PRO-12)	Combination of Tinetti balance score, Tinetti total score and ML trunk short term Lyapunov exponent: AUC = 0.760, Sens = 0.80, Spec = 0.7 ML trunk short term Lyapunov exponent alone is found insufficient for distinguishing groups. ML trunk short term Lyapunov exponent comparing non-fallers and multiple-fallers. AUC = 0.673; Sens: 0.53; Spec: 0.85	Performance metrics for Tinetti scores presented as suggested in previous reviews.
[48]	56/86 (RE-12)	Standing with feet together sway index: Sens = 78.6%; Spec = 75.7%; AUC = 0.84 (95% CI: 0.75–0.92) Standing with one foot in front sway index: Sens = 82.1%; Spec = 77.7%; AUC = 0.90 (95% CI: 0.82–0.97)	95% CIs for all AUCs presented. Performance metrics of CLIN BBS presented (both aspects suggested in previous reviews)

**Table 10:** Statistical analyses on the sensor-based classification models’ performance in classifying older individuals according to their risk of falling. NPV = Negative Predictive Value, PPV = Positive Predictive value.

Ref No.	No. of Fallers/No. of Participants (Faller/Non-Faller Labelling Method)	Classification Models/Algorithms Included in Discrimination Method	Model Validation Method	Methods’/Models’ Performance in Discriminating Groups with Different Level of Fall Risk (Fallers/Non-Fallers)	Comment
[20]	15/20 (RE-6, CLIN)	4 machine learning algorithms: NB, RBNC, KNN, SVM	Leave-one-out CV	NB: Sens = 1; Spec = 1; Err = 0 for 4 combinations of selected features RBNC: Sens = 1; Spec = 0.8; Err = 0.05 for 1 selected feature KNN: Sens = 0.93; Spec = 0.8; Err = 0.05 for 1 selected feature combination SVC: Sens = 1; Spec = 0; Err = 0.25 for 1 selected feature combination	Small sample Very high-performance metrics (authors conclude that NB probably is over fitted)
[23]	19/46 (PRO-12)	Logistic regression models (SENSOR, CONV)	Ten-fold CV (x10 times)	SENSOR: Acc = 70%; Sens = 58%; Spec = 78%; NPV = 72%; PPV = 65%; Brier score = 0.21; AUC = 0.72 CONV: Acc = 72%; Sens = 68%; Spec = 74%; NPV = 77%; PPV = 65%; Brier score = 0.20; AUC = 0.74	Small sample Performance metrics of clinical assessment tools provided (as suggested in previous reviews)
[27]	Number of fallers not reported in RE-60 and PRO-24 data samples/RE-60: 909 PRO-24: 259 Val: 55 (non-fallers)	Regularized discriminant classifier algorithms (Cross-sectional, prospective)	Ten-fold CV (x10 times) and validation using independent data set with healthy control subjects	1. Ten-fold CV (×10 times) Cross-sectional model (RE dataset): Acc = 70.02%; Sens = 47.73%; Spec = 84.72%; PPV = 70.14%; NPV = 69.19%; AUC = 0.67 Prospective model (PRO dataset): Acc = 76.27%; Sens = 57.20%; Spec = 83.63%; PPV = 59.86%; NPV = 82.54%; AUC = 0.69 2. Validation using independent datasets with healthy older adults Cross-sectional model (CS1 + CS2 non-faller): Acc = 94.11% PRO-model (CS1 + CS2 non-faller): Acc = 79.38%	Validation both by CV and by use of independent datasets (as suggested in previous reviews) Validation with independent dataset: Cross-sectional single task Acc > 81%
[29]	24/100 (RE-6)	3 machine learning algorithms: multi-layer perceptron NN, NB, SVM	Hold-out method (75% training set and 25% test set). Derived using either single task data or dual task data	Best fall risk classification model based on single task i.e., walk without cognitive load: 4 models with identical performance (SVM degree 2—using data from insoles-pelvis. SVM degree 3—using data from insoles-head-pelvis, NN 9 nodes—using data from insoles-pelvis, and NN 20—using data from insoles-head-pelvis-left shank): Acc = 84.0%; F1-score = 0.600; MCC = 0.521 Best fall risk classification model based on dual task (DT), i.e., walk with cognitive load: 1 SVM degree 1 model using data from insoles and pelvis: Acc = 80.0%; Sens = 100.0%; Spec = 73.7%; PPV = 54.5%; NPV = 100.0%; F1 = 0.706; MCC = 0.634 Comparison of 10 best ST models and 10 best DT models, all but one ST model outperformed the DT models.	Hold-out method used for model validation (preferred over CV in [9].) Single task data models: Best Acc > 81%
[32]	33/71 (RE-12, at least 2 falls)	4 machine learning algorithms (Ada Boost, SVM, bag, NB)	Stratified two-fold CV (×20 times)	Machine learning algorithms using features from Daily-Living Transitions: AdaBoost (mean number of (no) features = 18.25): Acc = 87.90%; Sens = 88.84%; Spec = 87.22% SVM (mean no features = 25.50): Acc = 90.64%; Sens = 89.23%; Spec = 91.66% Bag (mean no features = 10.25): Acc = 87.09%; Sens = 83.84%; Spec = 89.44% NB (mean no features = 19.10): Acc = 87.74%; Sens = 78.46%; Spec = 94.44% Machine learning algorithms using features from Daily-Living Transitions and functional laboratory tests: Ada Boost (mean no features = 13.55): Acc = 90.16%; Sens = 87.50%; Spec = 91.94% SVM (mean no features = 22.60): Acc = 91.00%; Sens = 88.75%; Spec = 92.50% Bag (mean no features = 16.05): Acc = 87.16%; Sens = 85.41%; Spec = 88.33% NB (mean no features = 15.65): Acc = 90.66%; Sens = 83.33%; Spec = 95.50% Machine learning algorithms using features from functional laboratory tests: AdaBoost (mean no features = 2.60): Acc = 70.00%; Sens = 56.66%; Spec = 78.88% SVM (mean no features = 1.70): Acc = 70.66%; Sens = 42.90%; Spec = 89.16% Bag (mean no features = 2.60): Acc = 72.50%; Sens = 68.75%; Spec = 75.00% NB (mean no features = 2.05): Acc = 70.66%; Sens = 44.16%; Spec = 88.33%	Authors find that features extracted from daily life can distinguish better between fallers and non-fallers than features from functional laboratory tests. The discrimination was only slightly improved by combining the features. Acc for model using data from Daily Living transitions > 81%
[36]	11/22 (RE-12)	FRE_sensor_—regularized discriminant model (sensor data) FRE_clin_—logistic regression model (clinical data) FRE_combined_—classifier combined theory	Classification performance: Leave-one out CV FRE_sensor_ features and model selection: Ten-fold CV (×10 times) Validation of FRE_sensor_ using independent dataset	Classification performance using leave-one-out CV: FRE_combined_: Acc = 68.48%; Sens = 68.36%; Spec = 68.57%; PPV = 61.11%; NPV = 75.00% FRE_sensor_: Acc = 66.82%; Sens = 74.01%; Spec = 61.63%; PPV = 58.22%; NPV = 76.65% FRE_clin:_: Acc = 58.53%; Sens = 35.93%; Spec = 78.90%; PPV = 54.55%; NPV = 63.61% Validation of FRE_sensor_ using independent dataset with community-dwelling older adults with 95% CI in []: Acc = 72.70 [54.12–91.34] %; Sens = 90.91 [78.90–100.0] %; Spec = 54.50 [33.69–75.31] %; PPV = 66.67 [46.97–86.37] %; NPV = 85.71 [71.09–100.0] %	Independent validation of clinical, sensor and combined FRE classifier models: FRE_sensor_ model validated on independent dataset and 95% CIs for performance metrics provided (as recommended in previous reviews)
[46]	82/196 (RE-60)	6 Machine learning algorithms: SVM, BT, RF, DT, NB, logistic regression	Ten-fold CV (×10 times) Two sample *t*-test for comparing overall classification Acc.	SVM: Acc = 89.42 ± 4.82%; Sens = 92.67 ± 6.17%; Spec = 84.90 ± 8.68% BT: Acc = 87.09 ± 5.56%; Sens = 91.23 ± 6.71%; Spec = 81.37 ± 9.37% RF: Acc = 86.39 ± 5.41%; Sens = 92.23 ± 5.49%; Spec = 78.06 ± 10.63% DT: Acc = 81.64 ± 6.09%; Sens = 87.25 ± 7.56%; Spec = 73.29 ± 10.62% NB: Acc = 80.05 ± 6.11%; Sens = 87.91 ± 6.60%; Spec = 69.16 ± 11.80%) Logistic regression: Acc = 79.70 ± 6.37%; Sens = 87.24 ± 6.75%; Spec = 69.23 ± 11.94%	95% CIs provided for each performance metrics (as recommended in previous reviews). Two sample *t*-tests on overall classification Acc showed that a significantly higher Acc was achieved using SVM. Best Acc > 81%
[51]	24/40 (RE-3, non/once/multi)	Three-class classification model using features from wearables and/or RAI-HC: POM, and the machine learning algorithms DT and RF Binary classification model using features from wearables and/or RAI-HC: logistic regression, DT and RF	Leave-one-out CV	1. Three class classification (non-fallers, fallers, multiple-fallers): (a) best performance RF: Acc = 0.838 ± 0.199; Recall = 0.775 ± 0.233; Precision = 0.730 ± 0.259; F_1_ = 0.748 ± 0.248 (b) DT: Acc = 0.757 ± 0.221; Recall = 0.703 ± 0.254; Precision = 0.643 ± 0.275; F_1_ = 0.662 ± 0.266 (c) worst performance POM: Acc = 0.676 ± 0.170; Recall = 0.626 ± 0.195; Precision = 0.593 ± 0.195; F_1_ = 0.584 ± 0.191 2. Binary classification (non-fallers + fallers vs. multiple-fallers): (a) best performance RF: AUC = 0.894 ± 0.155; Acc = 0.892 ± 0.160; Recall = 0.908 ± 0.135; Precision = 0.928 ± 0.106; F_1_ = 0.888 ± 0.166 (b) DT: AUC = 0.858 ± 0.160, Acc = 0.838 ± 0.200; Recall = 0.869 ± 0.154; Precision = 0.851 ± 0.226; F_1_ = 0.829 ± 0.218 (c) worst performance logistic regression: AUC = 0.838 ± 0.234; Acc = 0.703 ± 0.172; Recall = 0.676 ± 0.200; Precision = 0.657 ± 0.281; F_1_ = 0.626 ± 0.231 3. Binary classification (non-fallers vs. fallers + multiple-fallers): (a) best performance RF: AUC = 0.865 ± 0.125; Acc = 0.865 ± 0.132; Recall = 0.865 ± 0.125; Precision = 0.908 ± 0.094; F_1_ = 0.853 ± 0.139 (b) DT: AUC = 0.842 ± 0.229, Acc = 0.865 ± 0.192; Recall = 0.851 ± 0.213; Precision = 0.886 ± 0.202; F_1_ = 0.849 ± 0.214 (c) worst performance logistic regression: AUC = 0.743 ± 0.251; Acc = 0.784 ± 0.224; Recall = 0.766 ± 0.232; Precision = 0.778 ± 0.255; F_1_ = 0.755 ± 0.246	95% CI provided for each performance metrics (as recommended in previous reviews). Pruning used in training of DT models to avoid overfitting. Acc > 81%

## Data Availability

Not applicable.

## Abbreviations

The following abbreviations have been used in the article:

Acc	Accuracy	MML	Minimum Message Length
accel	Accelerometer	NEAT	Neuro evolution of augmenting topologies
Ada boost	Adaptive boosting	NB	Naïve Bayes(ian)
ADL	Activity in daily life	NN	Neural networks
ANOVA	Analysis of variance	NPV	Negative Predictive Value
ANCOVA	Analysis of co-variance	PLS-DA	Partial least square discriminatory analysis
AP	Anterior-posterior	POM	Proportional odds models
AUC	Area Under Curve (Operating Characteristics-curve)	PPV	Positive Predictive Value
Bag	Bootstrap aggregation	PRO	Prospective falls occurrence (followed number indicating period in months for prospective falls occurrence)
BBS	Bergs Balance Scale	QTUG	Quantitative TUG (a commercial tool)
BMI	Body Mass Index	RAI-HC	Resident Assessment Instrument—Home care
BST	Biometric Signature Trajectory	RBNC	Radial basis function network classifier
BT	Boosted tree	RCME	Refined composite multiscale entropy
C-GAITS	Comprehensive Gait Assessment using Inertial sensor	RE	Retrospective falls history (followed number indicating time period in months for falls history)
CI	Confidence intervals	RF	Random forest
CLIN	Clinical assessment methods	RMPE	Refined multiscale permutation entropies
COG	Center of gravity	RMS	Root Mean Square
CS	Cross-sectional	ROC	Receiver operating characteristic
CV	Cross validation	SagAngVel	Angular velocity in the sagittal plane
DT	Decision tree	SD	Standard Deviation
DTW	Dynamic Time Warping	SEF	Spectral Edge Frequency
EM	Expectation Maximization	SEMI-SUP	Semi-supervised
Err	Error	Sens	Sensitivity
F1-score	Harmonic mean of precision and Sens	SFRA	Sensor-based fall risk assessment
FoF	Fear of Falling	6MWT	Six-Minutes Walking Test
FRE	Fall risk estimate	Spec	Specificity
FTSS	5 times Sit-to-stand test	SSI	Step Stability Index
GMM	Gaussian Mixture Models	SSQ	Simulator Sickness Questionnaire
gyro	Gyroscope	SUP	Supervised
HR	Harmonic Ratio	SVM	Support vector machine
ICC	Intra-class correlation coefficient	TUG	Timed Up and Go
IMF	Intrinsic Mode Function	UEF	Upper extremities’ function
KNN	K-nearest neighbor	UNSUP	Unsupervised
LDS	Local Dynamic Stability	UT	Upper trunk
MANCOVA	Multivariate ANCOVA	Val	Validation
MCC	Matthew’s Correlation Coefficient	VR	Virtual reality
MGC	Minimum ground clearance	VRHMD	VR head-mounted display
ML	Medio-lateral	VT	Vertical

## Appendix A

The appendix provides details to text provided in Section 2.3 Data Extraction.

**Table A1:** Template for data extraction.

Variables	Type of Collected Data
1. First author	Free text
2. Title	Free text
3. Journal	Free text
4. Publication year	2010–2020
5. Population of older people in sample	Patients/Community-dwelling/Residential home (or similar)/Other
6. Faller/non-faller labelling method	RE and/or PRO and/or CLIN or other. For CLIN and PRO, the number of months used for collection of RE and PRO was collected.
7. Number of participants 60 years in sample	Number
8. Proportion of single fallers in sample	Percentage (0–100%)
9. Proportion of multiple fallers in sample	Percentage (0–100%)
10. Assessment task monitored by wearable sensors	Free text
11. Degree of supervision during task	SUP/SEMI-SUP/UNSUP
12. Number of wearable sensors	Number
13. Type of wearable sensors	Free text
14. Wearable sensor position(s)	Free text
15. Number of sensor features	Number
16. Feature selection methods	Free text
17. Signal processing methods used to discriminate/classify older adults according to fall risk (a) Wearable sensor features or (b) Models/algorithms able to discriminate significantly between fallers/non-fallers with machine learning (c) Models/algorithms able to discriminate significantly between fallers/non-fallers without machine learning	(a) Free text or (b) Free text or (c) Free text
18. Number of fallers/number of participants	Number/Number
19. Discrimination/classification performance, either (a) Sensor features’ performance in discriminating groups with different level of fall risk (fallers/non-fallers) (b) Methods’/model’s performance in discriminating groups with different level of fall risk (fallers/non-fallers) (c) Sensor features’ performance metrics in classifying individuals as fallers/non-fallers. Only the highest performance metrics are reported. (d) Model’s performance metrics in classifying individuals as fallers/non-fallers. Only the highest performance metrics for each model are reported.	(a) and (b) Free text (c) and (d) Free text including performance metrics values
20. For studies using classification methods/models (performance extracted in 19 b and d): (a) Classification models/algorithms included in discrimination method (b) Model validation method (c) Comments (of study methodology in relation to recommendations of previous review)	Free text (if used)
21. For studies analyzing discriminatory performance (performance extracted in 19 (a)–(b): Number and type of features able to discriminate groups with different level of fall risk (fallers/non-fallers)	Free text

## Appendix B

This appendix provides complimentary graphs to the information provided in Section 3.2.

## Appendix C

This appendix provides complimentary information for the feature selection methods in Table 4, Table 5 and Table 6.

**Table A2:** Complimentary information for the feature selection methods in Table 4. COG = center of gravity, ICC = Intra-class correlation coefficient, SSQ = Simulator Sickness Questionnaire.

Ref No.	Feature Selection Methods
[19]	1. Assessment of intra-and inter- observer reliability for gait parameters (ICC, CV of standard error of measurement) 2. Assessment of whether each parameter differed significantly between fallers/non-fallers and between walks (**ANOVA** and ***t*-test, Wilcoxon-signed-rank** and **Kruskall–Wallis tests** for step time asymmetry) 3. Analysis of each gait parameters’ predictive value (**Stepwise logistic regression**: forward likelihood ratio) 4. Analysis of discriminate capacity **(ROC curve)**
[21]	1. Assessment of whether each parameter differed significantly between repetitions for each participant (**ANOVA)** 2. Assessment of whether each parameter differed significantly between fallers/non-fallers **(One-way ANOVA)**
[24]	1. Assessment if whether characteristics and gait variables differed significantly between fallers/non-fallers **(****independent *t*-tests or χ2 tests)** 2. Analysis of each variable and falls incidence (**Stepwise logistic regression**: forward stepwise selection from all variables that were significantly associated with falling), 3. Estimation of cut-off values for gait variables significantly associated with falling in logistic regression to predict falls **(ROC curve)**
[25]	1. Assessment of whether SSI differed significantly between obstacle negotiation and baseline for controls/fallers **(Wilcoxon Signed Rank)** 2. Assessment of whether SSI differed significantly between fallers/controls under s different walking conditions (**Mann**–**Whitney test**)
[26]	Assessment of whether each parameter differed significantly between fallers/non-fallers (**Two-sided Student’s *t*-test**)
[30]	1. Assessment of the abilities of median LDS of each individual parameter setting to distinguish fallers/non-fallers (**Univariate logistic regression** with a 10-fold CV and AUC) and testing significant discrimination ability of each parameter setting (bootstrapping with 2000 resamples) 2. Comparison the 3 algorithms’ ability to distinguish fallers/non-fallers of best performing parameter setting (**Univariate logistic regression** with a 10-fold CV and AUC) 3. Selection of the best performing model for the 3 algorithms (**Stepwise multivariate logistic regression** with stepwise backward feature selection)
[31]	Selection of RCME/RMPE metrics with highest discriminatory ability (**PLS-DA** with a backward feature selection)
[33]	1. Assessment of whether each turning parameter differed significantly between non-fallers, single fallers, recurrent fallers based on RE-12 (**One-way ANOVA**) 2. Assessment of whether turning parameters differed significantly between non-fallers/fallers based on PRO-6 (**One-way ANOVA**)
[34]	1. Assessment of significant differences in postural sway (RMS and NPL sway in both AP and ML directions) between balance tasks (**Friedman test**, **Wilcoxon signed ranks test** for post hoc pairwise comparisons) 2. Assessment of test-retest reliability of lower extremity muscle strength (ICC) 3. Analysis of association between postural sway and muscle strength with gait speed, number of comorbidities, grip strength and frailty index (**Spearman rank correlation**) 4. Assessment of whether body sway and lower extremity strength differed significantly between fallers/non-fallers **(Mann**–**Whitney U test)**
[35]	1. Assessment of univariate group difference in the demographics and the walking patterns of fallers and non-fallers and further to adjust significant demographic differences between fallers/non-fallers **(ANOVA, ANCOVA)** 2. Assessment of associations between demographic, health, sensorimotor, psychological, and cognitive factors with the gait/mobility assessments (**Pearson’s correlation** and **Partial Pearson’s correction** (after adjusting for sex, age))
[37]	1. Assessment of whether each demographic parameter and adverse health outcomes differed significantly between the UEF index defined groups (non-frail/frail) **(ANOVA)** 2. Analysis of the association between the UEF index with each health outcome (**Logistic regression** (for nominal health outcomes) and **ANOVA regression** (for continuous health outcomes))
[38]	1. Assessment of the SSQ-score pre-and post-exposure (**one-sample *t*-test** and **signed rank test**) 2. Assessment of variable-module’s test-retest reliability for in identifying differences between 5 experimental conditions and baseline conditions **(Paired *t*-test** and **Pearson’s correlation)**
[39]	Identification of key outcome measures with significant impact on faller status (faller/non-faller) **(Stepwise logistic regression**, **Pearson correlation** to check multi-collinearity between all outcome measure pairs)
[40]	1. Assessment of performance difference for TUG parameters between single task (ST), motor task (MT) and cognitive dual-task (CT) conditions (**MANCOVA**) 2. Assessment of the difference in individual parameters from the three TUG conditions between groups of fallers/non-fallers (**ANCOVA**)
[41]	1. Assessment of differences between fallers and non-multiple fallers (**two-sample *t*-test**, **Fisher’s exact test** (for gender), **Mann**–**Whitney-U test** (for age and BMI), **Wilcoxon’s signed-rank test** (for pairwise differences in the gait parameters from two contrasting terrains), **Benjamini-Hochberg adjustments** (to control for multiple comparisons)) 2. **Logistic regression, Benjamini-Hochberg adjustments** on *p*-values (to identify individual gait parameters that were associated with multiple falls)
[42]	1. Assessment of differences between fallers/non-fallers/multiple fallers in demographics and clinical and basic gait assessment (**Mann–Whitney U test**) 2. Analysis of each significant variable’s strength to predict falls (**Logistic regression** and **ROC curve**)
[43]	1. Assessment of differences in socio-demographic parameters and subjective questionnaires among three balance groups (**One-way ANOVA model**) 2. Assessment of differences in balance parameters between groups (**ANOVA** models for normally distributed data according to Shapiro–Wilk test, **Friedman test** with calculation of Cohen’s effect size for data not normally distributed according to Shapiro–Wilk test) 3. Analysis of association between changes in COG sway body sway and changes with local-control(slope) and central-control(slope-log) changes due to vibration (**linear Pearson correlations**)
[44]	Assessment of differences in gait parameters between low-risk/high-risk patients (Independent samples ***t*-test**)
[45]	Assessment of whether the amount of DTW difference between reference and elderly fallers differed significantly from DTW differences of elderly non-fallers and reference (**ANOVA**) (DTW measures similarity in BST between elderly fallers and a reference BST)
[47]	1. Assessment of normality of data (**Shapiro–Wilk test**) and of homoscedasticity (**Levene’s Test** of Equality of Variances) 2.Assessment of falls history (faller/non-faller) on Micro and Macro gait characteristics (**General linear modelling**) 3. For gait characteristics with a fall history interaction: Post hoc secondary analysis (**Tukey’s test)** to identify subgroups (fallers/non-fallers among OAs) that are significantly different from each other.
[48]	1. Calculation of sway index (**GMM, EM and MML algorithm**) 2. Assessment of differences between non-fallers/fallers/multiple fallers in standing sway indices and BBS (**ANOVA**) 3. Assessment of the performance of 4 sway indices and BBS in predicting (once- and multiple) fallers (**ROC-curve**)
[50]	1. Assessment of differences in demographics and gait parameters between genders (**unpaired *t*-tests/χ2 tests**) 2. Assessment of structural validity and sub-domain construct validity of the C-GAITS score (exploratory factor analysis using **Unweighted least squares** as extraction method) 3. Assessment of the internal consistency of the C-GAITS score (**Cronbach’s alpha coefficient**) 4. Assessment of correlation between C-GAITS score and walking speed (**Single linear regression**) 5. Assessment of association of C-GAITS score and subscale scores with variables (**Unpaired *t*-tests**)

**Table A3:** Complimentary information for the feature selection methods in Table 5.

Ref No.	Feature Selection Methods
[22]	1. Assessment of significant differences between fallers and non-fallers in gait velocity and SagAngVel variables (**Mann**–**Whitney Wilcoxon rank sum**) 2. Assessment of correlation of each SagAngVel value with BBS score, manual TUG time, and gait velocity (**Pearson’s correlation**)
[23]	Pre-processing of multi-parameter datasets prior to classification modelling using a wrapper feature selection algorithm to exclude parameters with low information (**wrapper subset evaluator**, employing the **simple logistic algorithm**)
[27]	**Sequential forward feature selection** within CV procedure
[36]	1. FRE_sensor_ feature selection by using **nested CV** 2. Validation of FRE_sensor_ using an independent dataset
[49]	1. Assessment of whether FRE_combined_ differed significantly between fallers/non-fallers (**one-way ANOVA**) 2. Assessment of whether mobility impairment scores (calculated from percentile scores of QTUG parameters grouped in five functional categories) (**one-way ANOVA**)

**Table A4:** Complimentary information for the feature selection methods in Table 6.

Ref No.	Feature Selection Methods
[20]	1. Assessment of significant differences between fallers and non-fallers in each feature (***t*-test using Holm correction, Behrens-Fisher test**) 2. Feature selection (Method in the **forward wrapper selection** algorithm family
[28]	1.Assessment of associations between functional stair performance and participants characteristics (**Spearman’s rank correlations**) 2.Assessment of post-hoc medians and interquartile ranges (IQR) of stair climbing performance for subgroups with different stair-ascent strategies (**Kruskal**–**Wallis**)
[29]	1. Assessment of the ability of models to classify fallers/non-fallers based (**Acc, F1-score, MCC**) 2. Ranking analysis for each model type: (a) NB models (*n* = 62), (b) SVM models (*n* = 155), (c) NN models (*n* = 155) 3. Ranking analysis of best performing models for NB, SVM, NN models (15 of each) and ClinAsses models (5)
[32]	1. Assessment of the ability of the entire feature set to discriminate between the groups of older fallers/non-fallers (4 **machine learning algorithms** in Matlab) 2.Assessment of the ability of the individual features to distinguish between older fallers/non-fallers (**Linear regression analysis**)
[46]	1. Assessment of whether each of the 155 outcome measures differed significantly between faller/non-faller groups (**two-sample *t*-tests**) 2. Assessment of discriminative power of each of the 38 measures (**ROC analysis**) 3. Building models to classify faller/non-faller by use of 6 supervised machine learning models incorporating the 38 significant measures as predictor variables and fall status as response variable 4. Validation of classification models (10-fold CV): Acc 5. Statistical analysis of overall classification accuracy of SVM and the 5 other models (**two-sample *t*-test**)
[51]	1. Wearable data tested for normality (**Shapiro–Wilks**) 2. Assessment of whether means (or median) of each variable differed significantly between groups non-fallers/once-fallers/multiple-fallers (**one-way ANOVA** or **Kruskal**–**Wallis H test**) 3. Assessment of differences between groups with repeated measurements of physical activity, heart rate, and night sleep (**two-way ANOVA**) 4. Building models and evaluating their performance for three-class and binary fall risk classification (POM, logistic regression, DT, RF) including (a) Removal of collinear variables (multicollinear test); (b) Ranking the variables (predictors) importance in fall risk classification (recursive feature algorithm in Caret R package); (c) Training models on wearable, RAI-HC, wearable + RAI-HC
